# Biosynthesis of the Selenium-Substituted [FeFe]-Hydrogenases

**DOI:** 10.1021/jacs.6c08167

**Published:** 2026-06-16

**Authors:** Xin Yu, Toby Woods, Yu Zhang, R. David Britt, Guodong Rao, Thomas B. Rauchfuss

**Affiliations:** † School of Chemical Sciences, University of Illinois, Urbana, Illinois 61801, United States; ‡ Department of Chemistry, 8789 University of California, Davis, California 95616, United States

## Abstract

The [FeFe]-hydrogenase from *Chlamydomonas reinhardtii* (*Cr*HydA1) has been prepared with Se in place of S at the [2Fe]_H_ active site by HydF-mediated maturation using [HFe_2_(μ-SeH)­(μ-Se)­(CN)_2_(CO)_4_]^2–^ ([**2**]^2–^) and the apoenzyme. Complex [**2**]^2–^ is a hydride—a rare case where a metalloselenol and the (naturally occurring) metallothiol [Fe_2_(μ-SH)_2_(CN)_2_(CO)_4_]^2–^—adopt different structures. The structure of [**2**]^2–^ was deduced based on ^1^H and ^77^Se NMR spectroscopy. From [**2**]^2–^, highly active *Cr*HydA1-Se_2_ can be efficiently produced using CH_2_O, but not serine, as the precursor to the azadiselenolate cofactor. EPR/ENDOR spectroscopic studies were corroborated with isotopically pure *Cr*HydA1-^77^Se_2_ produced by maturation with [Fe_2_[(μ-^77^SeCH_2_)_2_NH]­(CN)_2_(CO)_4_]^2–^. *Cr*HydA1-Se_2_ and native *Cr*HydA1 have distinct, but quite similar geometric and electronic structures and redox properties.

## Graphical abstract

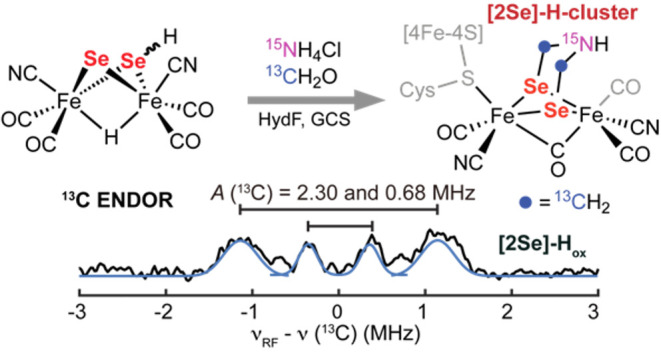

## Introduction

It is well recognized that selenium and sulfur share many chemical properties and that small amounts of Se are essential to many organisms, including humans.^1^ Several selenoproteins are known, most of which incorporate the 21st amino acid selenocysteine.^2^ Nonetheless, inorganic Se remains unknown in biochemistry, although further bioinformatics studies may be useful in this direction.^3^ Selenide has not been detected in any natural metal cluster^4^ despite the pervasiveness of Fe–S clusters^5^ and the stability of several salts of the type [Fe_4_Se_4_(SR)_4_]^2–^.^6^

Although unnatural, Se-for-S substitution can be induced in ferredoxins and Mo-based nitrogenase by treatment with selenide or selenocyanate.^7–11^ The [4Fe-4S] clusters in the Fe-protein of nitrogenase and [FeFe]-hydrogenase have also been replaced with a synthetic [4Fe-4Se] clusters.^12,13^ An Ni–Se bond is found in the [NiFeSe]-hydrogenases, which show some advantages over [NiFe]-hydrogenases in terms of catalytic profile.^14,15^ Naturally occurring selenium-substituted [FeFe]-hydrogenases have not been identified, although studies suggest that they might outperform their native counterparts as catalysts for hydrogen evolution.^16,17^

In this paper, we report a rare use of Se-for-S substitution to probe a biosynthetic pathway, that leading to the distinctive active site of the [FeFe]-hydrogenases. This active site has the formula [Fe_2_[(μ-SCH_2_)_2_NH]­(CN)_2_(CO)_3_]. Commonly referred to as [2Fe]_H_, this site features a collection of unusual cofactors including cyanide (CN^–^), CO, the azadithiolate ([(SCH_2_)_2_NH]^2–^ or adt), as well as an Fe–Fe bond. The biosynthesis of the [2Fe]_H_ has witnessed rapid advances in recent years motivated by the potentially useful catalytic properties of these enzymes.^18,19^ The biosynthesis, a process referred to as “maturation”, is mediated by three dedicated enzymes, the maturases HydG, HydE, and HydF.^18–27^ Our early work^28^ focused on Complex B, the product of HydG.^29^ In 2022, synthetic [Fe_2_(μ-SH)_2_(CN)_2_(CO)_4_]^2–^ was shown to allow reconstitution of HydA1 in the absence of HydG *and* HydE (Scheme 1).^30^ At present, the H-cluster can be maturated from [Fe_2_(μ-SH)_2_(CN)_2_(CO)_4_]^2–^ using a well-defined reaction cocktail.^26,31^ In addition to HydF and apo-HydA1, this cocktail contains the glycine cleavage system from the cellular C1 cycle, with serine and NH_4_^+^ serving as the carbon and nitrogen sources of the adt bridge, respectively. In the course of these studies we found that CH_2_O can be used instead of serine/SHMT to further simplify the defined maturation.^31^ These advances encouraged us to examine the biosynthesis targeting [Fe_2_[(μ-SeCH_2_)_2_NH]­(CN)_2_(CO)_3_] in the [2Fe]_H_ site of *Chlamydomonas reinhardtii* (*Cr*), giving *Cr*HydA1-Se_2_. The hydrogenase from *C. reinhardtii* is well suited for biosynthetic and associated spectroscopic analysis because it is readily overexpressed and, unlike most [FeFe]-hydrogenases, features only a single [4Fe-4S] cluster, the one attached to [2Fe]_H_ site.^32,33^ We expected that new mechanistic and spectroscopic insights would be provided using Se in place of S.

**Scheme 1:**
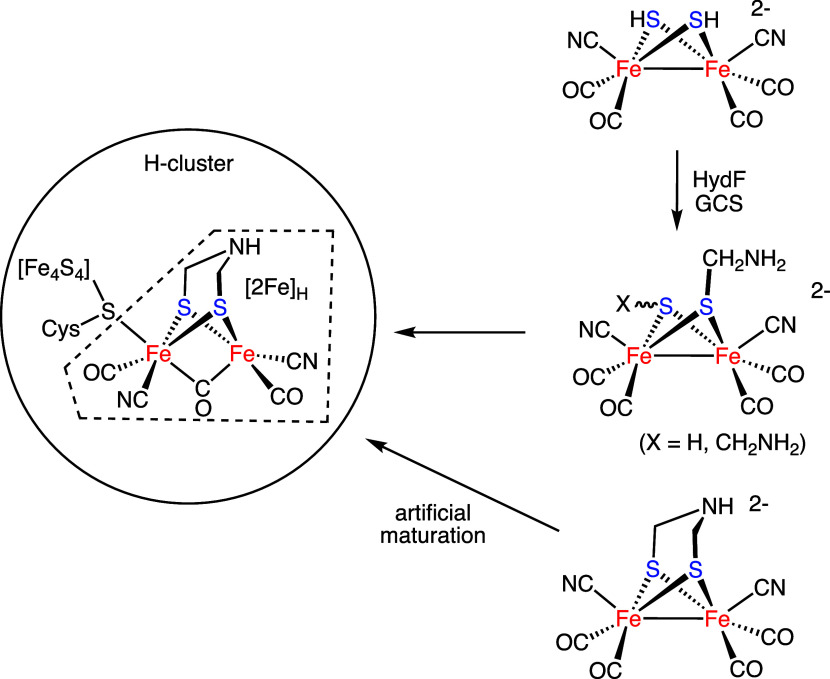
Late Stages in the Biosynthetic and Artificial Maturation of the H-Cluster

In 2013, it was reported that the [FeFe]-hydrogenase from *C. reinhardtii* could be reconstituted by combining the apoenzyme (i.e., the enzyme lacking [2Fe]_H_) with synthetic [Fe_2_[(μ-SCH_2_)_2_NH]­(CN)_2_(CO)_4_]^2–^.^34,35^ This process, called artificial maturation, facilitates the preparation of [FeFe]-hydrogenases by decoupling the complicated biosynthesis of [2Fe]_H_ from the production of a more routine iron–sulfur protein.^17^ In view of the recent availability^36^ of [Fe_2_[(μ-SeCH_2_)_2_NH]­(CN)_2_(CO)_4_]^2–^, we could apply artificial maturation to reconstitute apo-*Cr*HydA1 with the authentic Se analogue of the [2Fe]_H_ subsite, thereby corroborating the result of the biosynthetic route.

## Results and Discussion

### Part 1. [Fe_2_(μ-Se_2_)­(CN)_2_(CO)_4_]^2–^

The planned synthesis of [Fe_2_(μ-Se_2_)­(CN)_2_(CO)_4_]^2–^ was based on our preparation of [Fe_2_(μ-S_2_)­(CN)_2_(CO)_4_]^2–^ from Fe_2_(μ-S_2_)­(CO)_6_. That preparation uses two equiv of KN­(tms)_2_ (tms = SiMe_3_) to convert two CO ligands to two cyanide ligands.^37^ With respect to FT-IR characterization, dicyanation of Fe_2_(μ-S_2_)­(CO)_6_ shifts ν_COavg_ from 2040 to 1930 cm^–1^. Such a shift is consistent with strong electron-donating properties of CN^–^ vs CO.^37^ The analogous 2 KN­(tms)_2_ + Fe_2_(μ-Se_2_)­(CO)_6_ reaction however proceeded well only in the presence of 18-crown-6. Apparently, sequestering the K^+^ ion influences the course of the reaction. The [K­(18-crown-6)]^+^ salt [Fe_2_(μ-Se_2_)­(CN)_2_(CO)_4_]^2–^ ([**1**]^2–^) is a red-brown solid. The IR spectrum of this salt exhibits a single ν_CN_ band at 2078 cm^–1^ and ν_CO_ bands at 1971, 1920, and 1895 cm^–1^. The energies of these bands are within 6 cm^–1^ of those for the related [Fe_2_(μ-S_2_)­(CN)_2_(CO)_4_]^2–^.^37^ The ^13^C NMR spectrum is simple, displaying only resonances for the two kinds of diatomic ligands: δ 223 for *C*O and δ 147 for *C*N^–^.

The structure of [K­(18-crown-6)]_2_[**1**] was determined by X-ray crystallography (Figure 1). The cyanide ligands are diapical on the Fe_2_ framework, as is often observed in related sulfur systems.^38^ The K^+^ centers are bound to the FeC*N* groups as well as the crown ether. The crystallographic result shows that the Se–Se bond remains intact, being almost identical to the Se–Se bond of 2.293(2) Å in Fe_2_(μ-Se_2_)­(CO)_6_.^39^ We also had anticipated the possible formation of the diferrous [Fe_2_(μ-Se)_2_(CN)_2_(CO)_4_]^2–^.^40^

**Figure 1:**
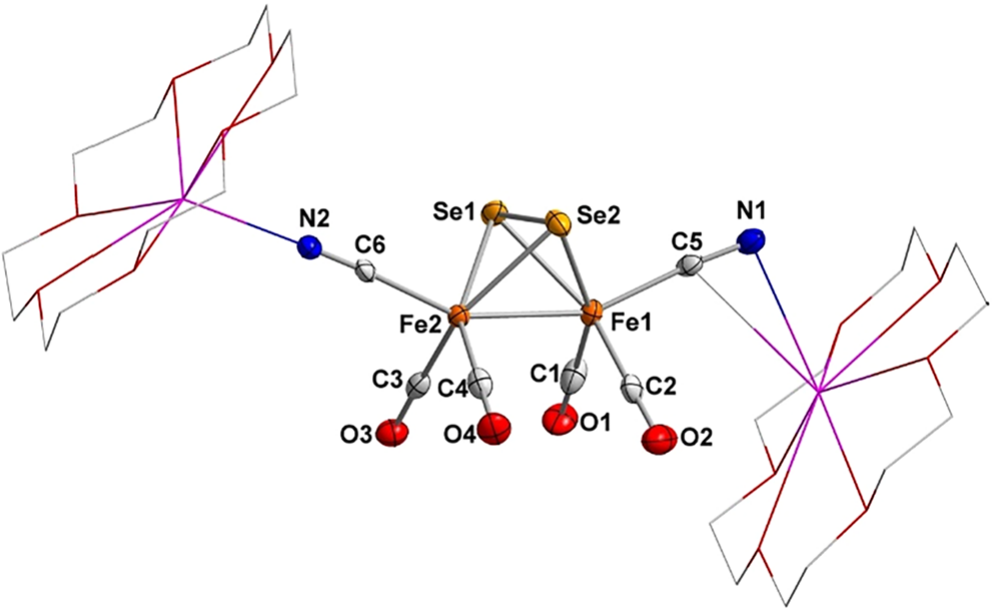
Structure of [K­(18-crown-6)]_2_[**1**] with H atoms omitted for clarity. Selected distances (Å): Fe1–Fe2, 2.5749(15); Fe1–Se1, 2.3887(15); Fe1–Se2, 2.3721(15); Fe2–Se1, 2.3942(14); Fe2–Se2, 2.3778(14); Fe1–C1, 1.753(14); Fe1–C2, 1.735(9); Fe1–C5, 1.939(8); Fe2–C3, 1.740(9); Fe2–C4, 1.752(9); Fe2–C6, 1.948(9); Se1–Se2, 2.3030(13). Angles (deg): Fe1–Se1–Fe2, 65.14(4); Fe1–Se2–Fe2, 65.65(4).

### Synthesis of [HFe_2_(μ-SeH)­(μ-Se)­(CN)_2_(CO)_4_]^2–^

The diselenol complex [Fe_2_(μ-SeH)_2_(CN)_2_(CO)_4_]^2–^ was the next target of our efforts. This compound would be a heavy analogue of [Fe_2_(μ-SH)_2_(CN)_2_(CO)_4_]^2–^, an intermediate in the biosynthesis of the [FeFe]-hydrogenases. The preparation of [K­(18-crown-6)]_2_[Fe_2_(μ-SeH)_2_(CN)_2_(CO)_4_] was attempted by treating a solution of [K­(18-crown-6)]_2_[**1**] with *N*,*N*′-bis­(trimethylsilyl)-4,4′-bipyridine ((tms)_2_-4,4′-bipy) in MeCN. As popularized by Mashima et al. for reductive dehalogenation reactions,^41^ this organosilicon reagent is a donor of two Me_3_Si· groups. We previously used this reagent to convert [Fe_2_(μ-S_2_)­(CN)_2_(CO)_4_]^2–^ into [Fe_2_(μ-SH)_2_(CN)_2_(CO)_4_]^2–^.^37^ When applied to the reduction of [**1**]^2–^, the Mashima reagent produced a homogeneous solution. ^1^H NMR analyses indicated a complicated set of products (Figure S7). The reproducibility of the spectrum suggested that the reaction produces an equilibrium mixture of several isomers. As shown below, these isomers can be assigned by correlating high- and midfield ^1^H NMR signals as well as the ^77^Se spectrum.

### ^1^H NMR Analysis of [HFe_2_(μ-SeH)­(μ-Se)­(CN)_2_(CO)_4_]^2–^

The following analysis assumes that the main products are diferrous species of the type [(μ-H)­Fe_2_(μ-SeH)­(μ-Se)­(CN)_2_(CO)_4_]^2–^ ([**2**]^2–^). The ^1^H NMR spectrum shows five singlets near δ −1, four of which are assigned to Se*H* (Figure 2). Four of these signals show shoulders characteristic of coupling to ^77^Se, which has 7% natural abundance. The values of ^1^ *J*(^77^Se,^1^H) are ∼30 Hz, which is typical.^42,43^ In addition to the Se*H* signals near δ 0, major singlets are observed at δ −12.00, −16.30, −16.43, and −20.49 (Figure 2). These signals are assigned to Fe-*H*-Fe sites. Such high-field shifts are well precedented, e.g., by [(μ-H)­Fe_2_[(μ-SCH_2_)_2_NH]­(CN)_2_(CO)_4_]^−^, which shows a hydride signal at δ −19.2.^36^ These four high field signals do *not* exhibit coupling to ^77^Se. Signals near δ 0 are also seen for related sulfhydryl complexes Fe_2_(μ-S*H*)_2_(CO)_4_(PPh_3_)_2_,^44^ [Fe_2_(μ-S*H*)_2_(CN)_2_(CO)_4_]^2–^,^30^ and [Fe_2_(μ-S*H*)­(μ-SR)­(CN)_2_(CO)_4_]^2–^.^37^

**Figure 2:**
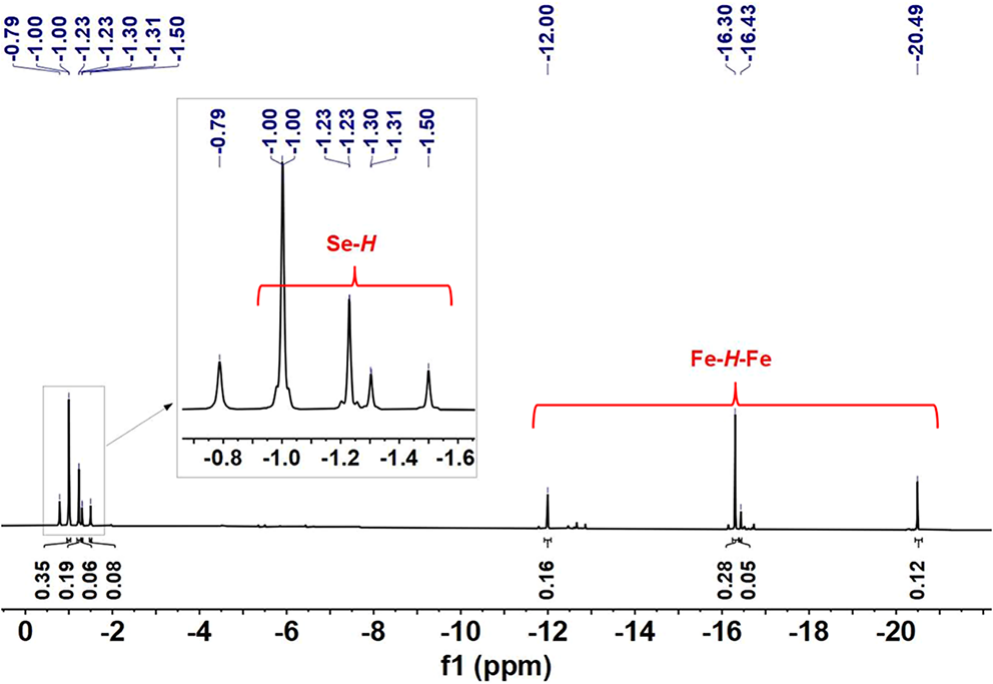
^1^H NMR spectrum (CD_3_CN) of [K­(18-crown-6)]_2_[**2**] in the high field region. The signals in the range δ −1 to −1.5 are assigned to Se*H*. The signals in the range δ −12 to −21 are assigned to Fe-*H*-Fe. The signal at δ −0.8 is unassigned.

To more fully assign the spectra, two further assumptions are invoked. First, with a pair of low-spin octahedral Fe­(II) sites, the various isomers would be stereochemically rigid on the NMR time scale. This situation is similar to that for a variety of bioctahedral Fe­(II)_2_(μ-SR)_2_ complexes, e.g., the propanedithiolate (pdt) complexes [(μ-H)­Fe_2_(μ-pdt)­(CN)_2_(CO)_4_]^−^ and [(μ-SMe)­Fe_2_(μ-pdt)­(CO)_4_(PMe_3_)_2_]^+^.^45,46^

Second, we assume rapid intramolecular proton exchange between μ-SeH and μ-Se sites. Fast exchange would be consistent with the ∼1000× increased acidity of Se–H vs S–H bonds.^5,47^ Because of this fast exchange, equatorial and axial SeH groups rapidly interconvert *and* the proton rapidly hops to the adjacent Se (Scheme 2).

**Scheme 2:**
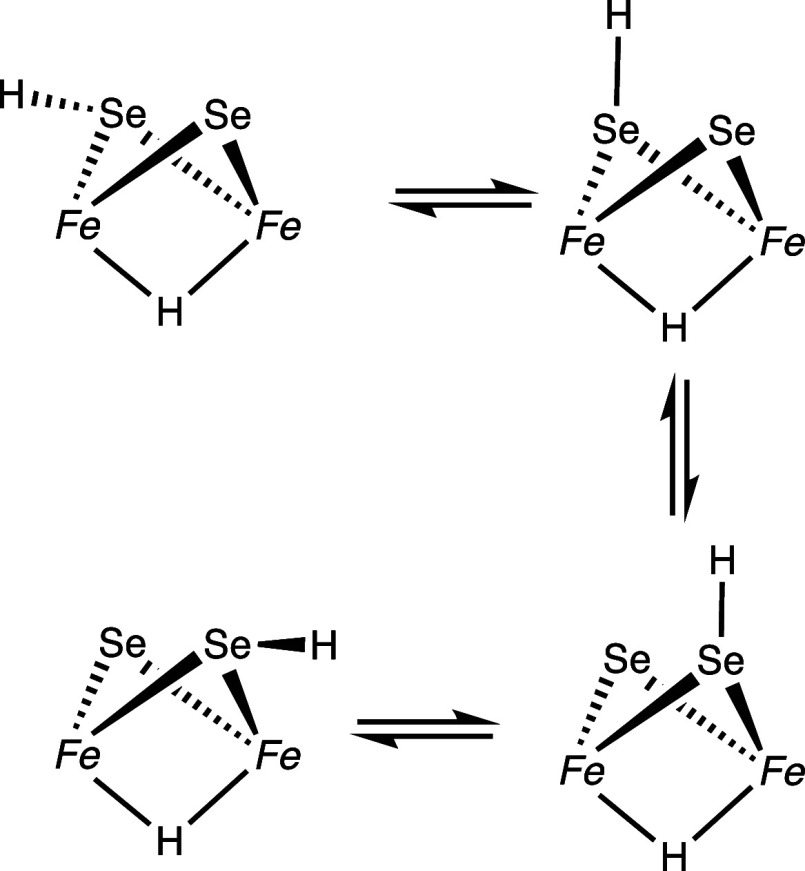
Proposed Intramolecular Proton Hopping Process That Is Consistent with the ^1^H and ^77^Se NMR Data (*Fe* = Fe­(CN)­(CO)_2_^–^)

The above analysis is further supported by the observation that the lower field and higher field signals are of comparable intensity, i.e., Se*H* and Fe*H*Fe groups are paired within the same complexes.

### ^77^Se NMR Analysis of [HFe_2_(μ-SeH)­(μ-Se)­(CN)_2_(CO)_4_]^2–^

The ^1^H NMR analysis above was complemented by ^77^Se NMR, which reinforced the assignments. The ^1^H–^77^Se correlation spectrum revealed the presence of four species (isomers), as suggested by the ^1^H NMR spectrum (Figure 4). In two of the isomers, the Se sites are equivalent. The Se centers are nonequivalent in the other *two isomers* (Figure 3). In the two with equivalent ^77^Se centers (δ −209, −284), the positions of the cyanide ligands must be symmetry-related with respect to the Fe_2_Se_2_ core, i.e., diapical, or cis to both Se atoms (*C*_2*v*_) or *trans*-dibasal or trans, cis to the Se atoms (*C*_2_). In each of these isomers, the μ–Se-H and μ-Se sites are made equivalent by rapid exchange. These equivalent isomers account for four of the ^1^H NMR signals (Figure 3). In the two species with nonequivalent ^77^Se centers, the two FeCN groups are apical-basal (*C*_1_) and *cis*-dibasal (*C*_s_). In these stereoisomers, the Se sites are nonequivalent regardless of exchange between Se and SeH sites. These isomers show ^1^H NMR signals at δ −1.03 and −1.26 and δ −16.3 and −20.5, respectively for Se*H* and Fe*H*Fe.

**Figure 3:**
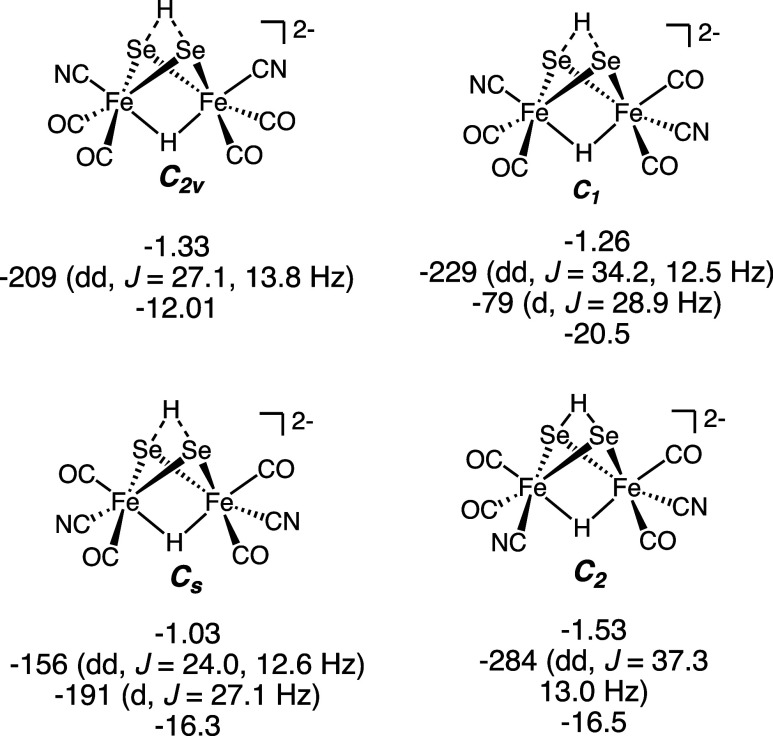
Four isomers of [**2**]^2–^ showing the acidic proton between the Se centers to emphasize its lability. The *C*_2*v*_ and *C*_2_ isomers are “symmetric” with time-averaged Se sites. The *C*_s_ and *C*_1_ isomers have nonequivalent Se sites. ^1^H NMR chemical shifts are shown for δ_SeH_ and δ_Fe*H*Fe_ in the top, and bottom rows, respectively. ^77^Se NMR chemical shifts are shown in the middle row(s).

**Figure 4:**
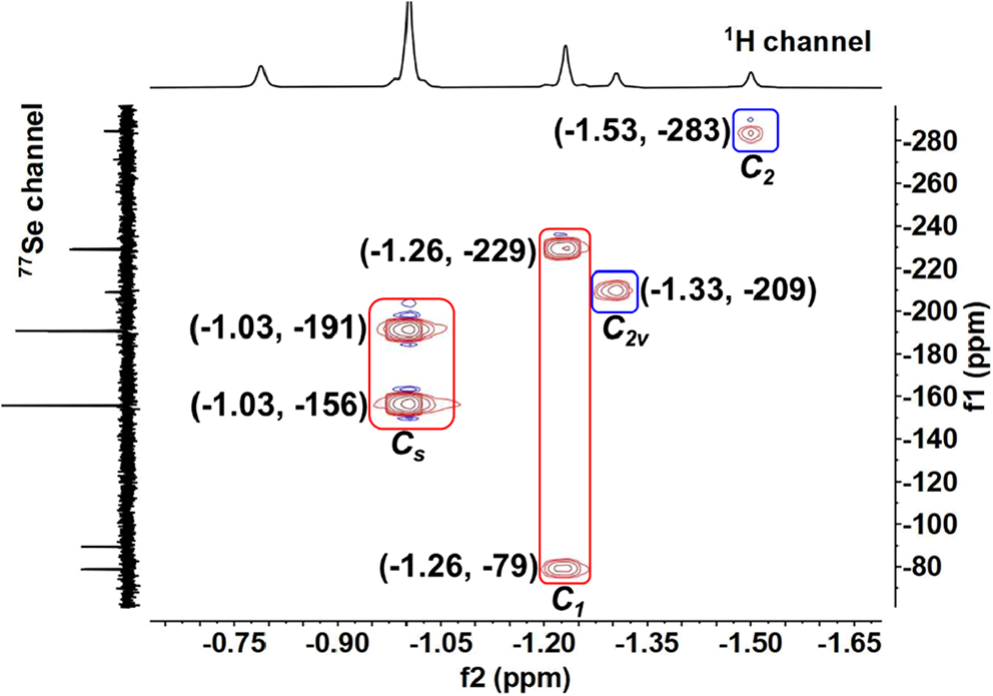
^1^H–^77^Se HSQC spectrum of [K­(18-crown-6)]_2_[**2**].

### Dehydrogenation of [HFe_2_(μ-SeH)­(μ-Se)­(CN)_2_(CO)_4_]^2–^

Treating a CD_3_CN solution of [**2**]^2–^ with two equiv of TEMPO cleanly gave [**1**]^2–^, as verified by ^1^H, ^13^C, and ^77^Se NMR spectroscopies (Figures S11–S13). We had previously reported that TEMPO serves as an H-atom abstracting reagent, effecting the dehydrogenation of Fe_2_(μ-SH)_2_(CO)_4_(PPh_3_)_2_.^44^ Thus, even though [**2**]^2–^ consists of multiple species, oxidation cleanly gives a single product (Scheme 3).

**Scheme 3:**
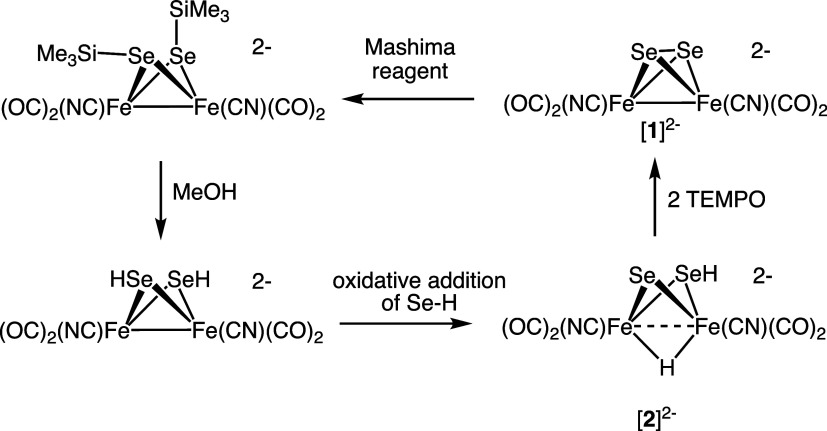
Proposed Pathway Connecting [**1**]^2–^ and [**2**]^2–^

### H/D Exchange of [HFe_2_(μ-SeH)­(μ-Se)­(CN)_2_(CO)_4_]^2–^ with CD_3_OD

When a CD_3_CN solution of [K­(18-crown-6)]_2_[**2**] was allowed to stand for 80 min in the presence of excess CD_3_OD, both the Se–*H* and hydride signals disappeared from the ^1^H NMR spectrum. In a similar experiment, we monitored the ^1^H NMR signal for a solution of [K­(18-crown-6)]_2_[**2**] in 0.6 mL of CD_3_CN containing 40 equiv of CD_3_OD. Within 10 min, the Se–*H* signals had disappeared, but the hydride remained almost unchanged (Figure S14). After 80 min, solvent was removed, and the residue was examined by ^2^H NMR spectroscopy as a MeCN solution. Signals were observed for Se–D and Fe–D–Fe (Figure S15).

The reactivity of [**2**]^2–^ with TEMPO also supports the dynamic structure of this selenol-hydride. We propose that a key step in the H/D exchange mechanism between [**2**]^2–^ and CD_3_OD involves a dynamic process via “[Fe_2_(μ-SeH)_2_(CN)_2_(CO)_4_]^2–^” as a postulated intermediate (Scheme 4).

**Scheme 4:**
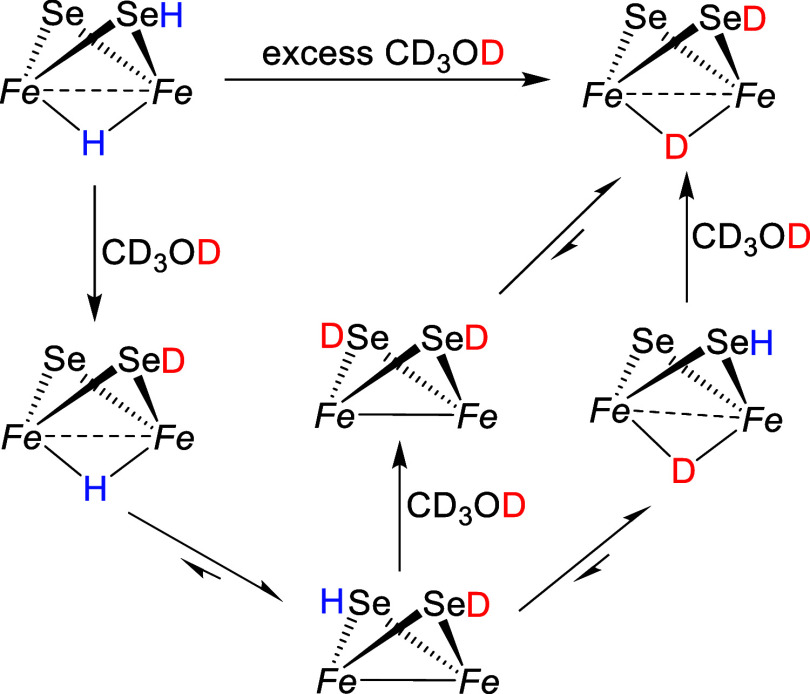
Proposed Mechanism of the H/D Exchange in [**2**]^2–^

#### Part 2. Defined Maturation of *Cr*HydA1-Se_2_ Using Fe_2_Se_2_ Precursors

We attempted the maturation of *Cr*HydA1-Se_2_ using [**2**]^2–^ under conditions standardized for [Fe_2_(SH)_2_(CN)_2_(CO)_4_]^2–^ (see SI for details).^31^ To our initial surprise, the defined maturation yielded only a low activity product (Figure 5). The H_2_ evolution activity of the maturated product is ∼3 μmol/mg/min, which is only 1.4% of correctly assembled *Cr*HydA1-Se_2_ (vide infra). Instead, the major *Cr*HydA1 product is a [4Fe-4S]_H_-[Fe_2_Se_2_(CO)_3_(CN)_2_] species, or [2Se]-H_Δbridge_. This same species, which can be formed by simply incorporating [**2**]^2–^ into apo-*Cr*HydA1, has no H_2_ evolution activity. We had previously observed the sulfur version of H_Δbridge_ in the defined maturations using [Fe_2_(SH)_2_(CN)_2_(CO)_4_]^2–^ under similar conditions.^31^ Similar to the H-cluster, [2Se]-H_Δbridge_ can be poised in the mixed-valence Fe^I^Fe^II^ state by the mild oxidant thionine, giving characteristic H_ox_-like and H_ox_-CO-like EPR signals, with *g* = [2.108, 2.059, 1.995] and *g* = [2.021, 2.010, 1.984], respectively. In addition, the H_ox_-CO like signal also underwent photolysis to generate the H_ox_-like signal, as does the H-cluster.^48,49^

**Figure 5:**
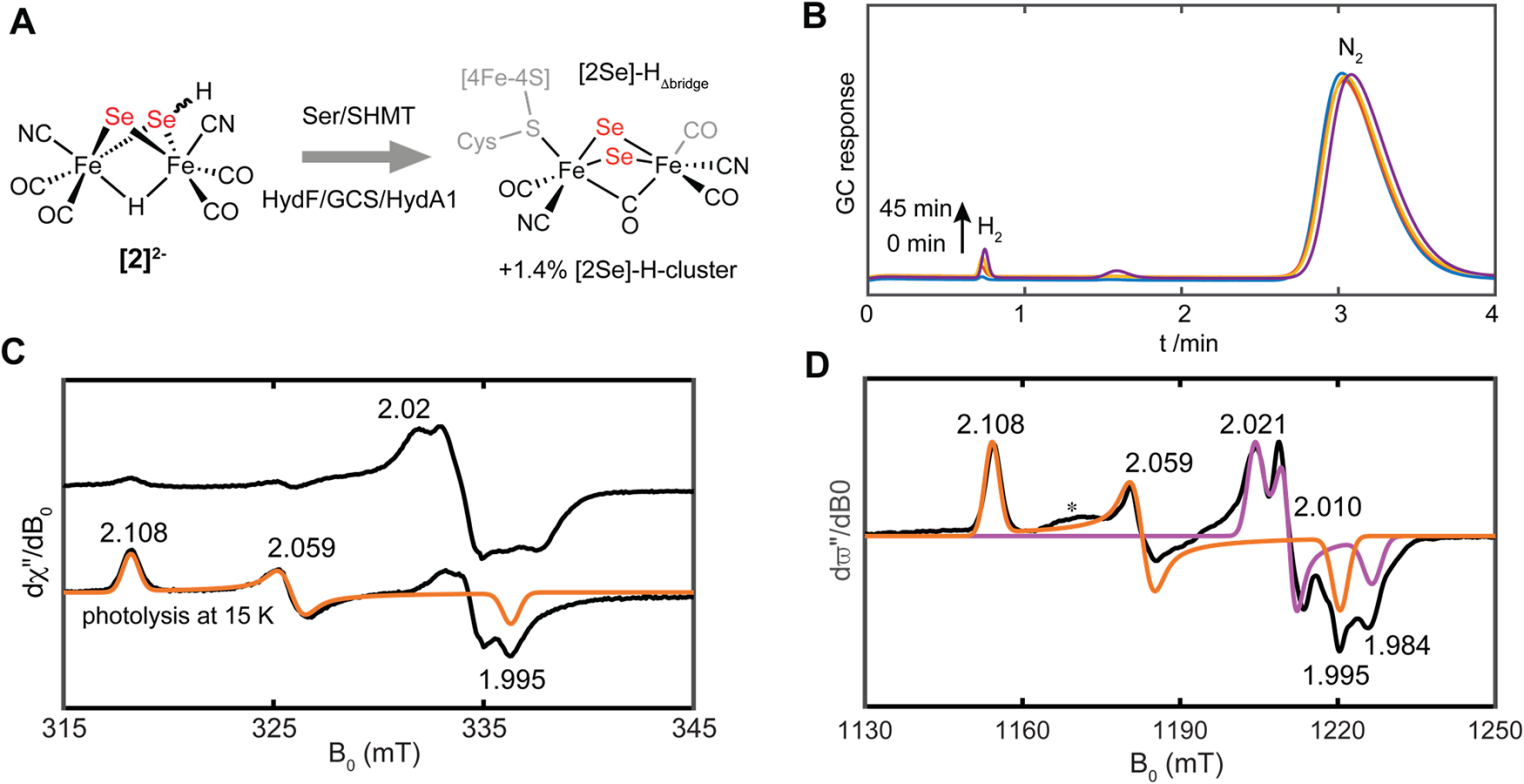
Maturation of *Cr*HydA1-Se_2_ using [**2**]^2–^ with serine as the adt carbon source. (A) Scheme of the maturation reaction. (B) GC time course of the H_2_ evolution assay of the maturation product. N_2_ is the carrier gas in GC assays. Note the area ratio between H_2_ and N_2_ compared to that in Figure 6 (vide infra). (C) X-band EPR spectra (15 K, 0.02 mW) of [Se]_2_-H_Δbridge_ before and after photolysis at 15 K for 20 min. (D) Q-band pseudomodulated field-sweep EPR spectrum (15 K, τ = 300 ns) of the photolyzed sample in C. Simulation parameters: for the H_ox_-like signal, *g* = [2.108, 2.059, 1.995], *g*Strain = 0.005; for the H_ox_-CO like signal, *g* = [2.021, 2.010, 1.984], *g*Strain = 0.005. The asterisk (*) denotes an unknown signal not included in the simulation.

The differing outcomes for maturation using [**2**]^2–^ vs the analogous (SH)_2_ precursor may be attributed to the differing redox behavior of sulfides and selenides in the glycine cleavage system. Selenolipoylated H-protein of the glycine cleavage system was synthesized and characterized previously.^50^ It was found that the diselenide form of selenolipoylated H-protein was a poor substrate, and that the rate of the overall glycine cleavage reaction with selenolipoylated H-protein was <1% of that with lipoylated H-protein. While the exact mechanism by which the adt bridge is installed onto the diiron core remains uncertain, these observations may provide some basis to explain our maturation results.

Strikingly, more successful maturation using [**2**]^2–^ was achieved with CH_2_O as the carbon source in place of serine and SHMT (Figure 6). Formaldehyde is known to spontaneously condense with tetrahydrofolate to generate methylenetetrahydrofolate, the biological equivalent of “active formaldehyde”.^51,52^ A higher concentration of formaldehyde likely drives the otherwise unfavorable transfer of the aminomethyl moiety to Fe_2_(SeH)_2_. The resulting *Cr*HydA1-Se_2_ is highly active, with H_2_ evolution activity of ∼220 μmol/mg/min (U), which is higher than that of the heterologously expressed (150 U) or in vitro maturated *Cr*HydA1 (175 U),^31,53^ albeit lower than the native CrHydA1 enzyme isolated from *C. reinhardtii* (935 U) or that expressed in *Clostridium acetobutylicum* (625 U).^54,55^ The EPR spectrum of thionine-oxidized *Cr*HydA1-Se_2_ shows primarily the H_ox_-CO signal (*g* = [2.059, 2.024, 2.017]), but the H_ox_ species can be generated via photolysis at 15 K (*g* = [2.105, 2.056, 2.003]). The exact same H_ox_ and H_ox_-CO EPR signals were detected in a sample of apo-*Cr*HydA1 reconstituted with [**3**]^2–^. This corroborates the correct assembly of the [(SeCH_2_)_2_NH]^2–^ cofactor.

**Figure 6:**
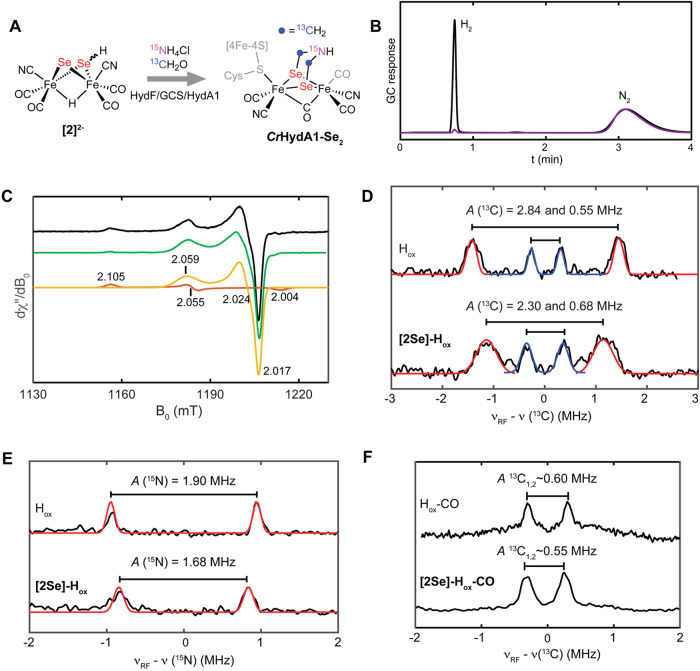
Maturation of *Cr*HydA1-Se_2_ using [K­(18-crown-6)]_2_[**2**] with ^13^CH_2_O as the adt carbon source. (A) Scheme of the maturation reaction. (B) GC of the H_2_ evolution assay (at 45 min) of the maturation product (black trace). N_2_ is the carrier gas in GC assays. The purple trace is the 45 min GC trace from Figure 5B. (C) Q-band pseudomodulated field-sweep EPR spectrum (15 K, 0.02 mW) of maturated [2Se]-H-cluster (black trace) compared with that maturated with [**3**]^2–^. Simulation parameters: for the H_ox_, *g* = [2.105, 2.055, 2.004], *g*Strain = [0.007, 0.006, 0.008]; for the H_ox_-CO like signal, *g* = [2.059, 2.024, 2.017], *g*Strain = [0.014, 0.008, 0.0035]. (D) Q-band ^13^C Mims ENDOR spectra of [2Se]-H_ox_ and H_ox_ recorded at *g* = 2.103 showing the two magnetic inequivalent ^13^C in the adt bridge. (E) Q-band ^15^N Mims ENDOR spectra of [2Se]-H_ox_ and H_ox_ recorded at *g* = 2.103. (F) Q-band ^13^C Mims ENDOR spectra of [2Se]-H_ox_-CO and H_ox_-CO recorded at their respective *g*_2_.

Further evidence for the successful maturation of *Cr*HydA1-Se_2_ from [**2**]^2–^ using CH_2_O in place of the GCS comes from ENDOR experiments. When using ^13^CH_2_O and ^15^NH_4_Cl in the maturation cocktail, the adt bridge of the resulting [2Se]-H-cluster is isotopically labeled. ^13^C Mims ENDOR spectrum recorded at *g* = 2.105, the *g*_1_ edge of [2Se]-H_ox_, reveals two sets of ^13^C hyperfine couplings, characteristic of the magnetically inequivalent adt carbons in the H-cluster.^56^ Based on previous quantum chemical studies,^57^ the carbon atom with the larger hyperfine coupling is assigned to the position cis to the distal CN^–^ ligand. We expect that the same assignment is applicable to CrHydA1-Se_2_. The ^13^C hyperfine values at this field position are 2.30 and 0.68 MHz, which are similar but distinct to those for H_ox_ (2.84 and 0.55 MHz, respectively^56^). The corresponding adt ^15^N hyperfine coupling of [2Se]-H_ox_ at this field is 1.68 MHz, which is, again, similar to but distinct from that in H_ox_ (1.90 MHz). In addition, ^13^C Mims ENDOR recorded at *g* = 2.02, *g*_2_ of [2Se]-H_ox_-CO, shows the overlapping ^13^C hyperfine coupling of both adt carbons of 0.55 MHz, which is comparable to the value in H_ox_-CO, 0.60 MHz. These ENDOR results provide solid evidence that Se-substituted H-cluster was formed under our maturation conditions. The EPR parameters of [2Se]-H-cluster are summarized in Table 1 together with those of the sulfur congener.

**Table 1:** EPR and ENDOR Parameters for Native and 2Se-Modified *Cr*HydA1

		*g*-factor	*A* ^13^C adt (MHz)	*A* ^15^N adt (MHz)
*Cr*HydA1-Se_2_	H_ox_	[2.105, 2.055, 2.004]	2.30 and 0.68^a^	1.68^a^
	H_ox_-CO	[2.059, 2.024, 2.017]	0.60^b^	-
	H_Δbridge_	[2.108, 2.059, 1.995]	-	-
*Cr*HydA1	H_ox_	[2.103, 2.044, 1.998]	2.84 and 0.55^a^	1.90^a^
	H_ox_-CO	[2.053, 2.009]	0.55^c^	-
	H_Δbridge_	[2.106, 2.051, 1.997]	-	-

a Measured at *g* = 2.103.

b Measured at *g* = 2.024.

c Measured at *g* = 2.009.

### Maturation of *Cr*HydA1-Se_2_ with [Fe_2_[(μ-^77^SeCH_2_)_2_NH]­(CN)_2_(CO)_4_]^2–^

As a final complement to this biosynthetic study, we reconstituted apo-HydA1 with [Fe_2_[(μ-^77^SeCH_2_)_2_NH]­(CN)_2_(CO)_4_]^2–^ ([^77^ **3**]^2–^) prepared from 99+% enriched ^77^Se. This isotopologue was prepared similarly to the unenriched complex and isolated as its (NEt_4_)^+^ salt.^36^ The approach is similar to that employed by Kertess et al.,^16^ but uses well-defined Fe_2_ reagent (i.e., [^77^ **3**]^2–^). Its FT-IR spectrum matched that of the unenriched sample, but the NMR spectra showed the distinctive effects of ^77^Se (*I* = 1/2). In particular, the ^13^C­{^1^H} NMR signal for the CH_2_ group in [^77^ **3**]^2–^ appears as a multiplet (ddd) at 38.06 ppm (*J*_Se–C_ = 78.4 Hz, *J*_Se–C_ = 19.7 Hz, *J*_H–C_ = 5.9 Hz), whereas the CH_2_ signal in the ^13^C­{^1^H} spectrum of [**3**]^2–^ appears as a singlet at the same chemical shift.

In terms of biosynthesis, [^77^ **3**]^2–^ readily inserts into apo-*Cr*HydA1 to yield highly active *Cr*HydA1-Se_2_. The *g* values of the oxidized sample containing [2Se]-H_ox_ and [2Se]-H_ox_-CO match well with those of the sample obtained from maturation using [**2**]^2–^ (Figure 7A and Table 1). In addition, ^77^Se labeling allows the first direct measurement of ^77^Se hyperfine couplings. For [2Se]-H_ox_, Davies ENDOR spectrum recorded at *g*_1_ reveals two sets of ^77^Se hyperfine couplings of ca. 6 and 12 MHz, respectively (Figure 7B). The presence of two inequivalent Se couplings is consistent with the inequivalent ^13^C hyperfine couplings of the adt moiety observed in both native *Cr*HydA1^56^ and *Cr*HydA1-Se_2_ (vide supra). Notably, both the ^13^C and ^77^Se hyperfine couplings differ by approximately a factor of 2. Extending the conclusions of previous quantum chemical studies,^57^ the Se atom with the larger hyperfine coupling can likewise be assigned to the position cis to the distal CN^–^ ligand.

**Figure 7:**
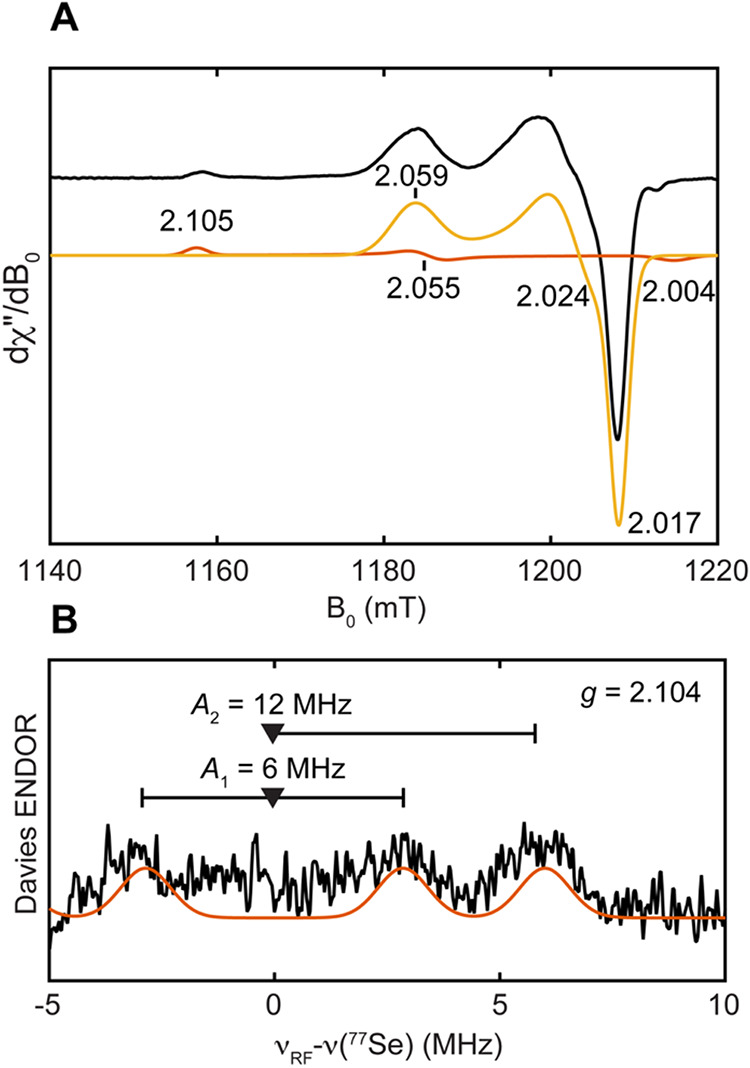
(A) Q-band pseudomodulated echo-detected EPR spectra of *Cr*HydA1-^77^Se_2_, simulated with the [2Se]-H_ox_ and [2Se]-H_ox_-CO components using parameters from Table 1. (B) Q-band Davies ENDOR spectrum of the same sample recorded at 1160 mT, *g* = 2.104. The frequency axis is normalized against the Larmor frequency of ^77^Se (9.47 MHz). The ν_–_ manifold of the 12 MHz hyperfine coupling is not shown since it would appear at a low frequency region (∼3 MHz) where the RF signal is too weak.

## Conclusions and Summary

The renowned hydrogenase *Cr*HydA1 has been prepared with Se in place of S at the [2Fe]_H_ active site. This achievement required the preparation of a suitable 2Fe-2Se precursor, which behaves differently from the native 2Fe-2S precursor and a maturation protocol that also differs from the natural biosynthesis.

Part 1 discloses a surprising finding: reduction of [Fe_2_(μ-Se_2_)­(CN)_2_(CO)_4_]^2–^ gives [HFe_2_(μ-SeH)­(μ-Se)­(CN)_2_(CO)_4_]^2–^, not [Fe_2_(μ-SeH)_2_(CN)_2_(CO)_4_]^2–^. This is an unusual case where a metalloselenol and a metallothiol adopt different structures.^57^ One can envision that [Fe_2_(μ-SeH)_2_(CN)_2_(CO)_4_]^2–^ tautomerizes to [**2**]^2–^ by transfer of one proton from Se to Fe. Evidence is presented in the form of H-D exchange and TEMPO-induced dehydrogenation, that the tautomerism is reversible.

At a fundamental level, [**2**]^2–^ provides a rare example of an Fe_2_–SeH complex. In classic work, Seyferth reported the LiBHEt_3_ reduction of Fe_2_(μ-Se_2_)­(CO)_6_,^58^ but the protonation of the implied product (Li_2_Fe_2_(μ-Se)_2_(CO)_6_) was not described.^59^ One might expect Fe_2_(μ-SeH)_2_(CO)_6_ to be highly acidic, thus the two cyanide ligands in [**2**]^2–^ elevate the p*K*_a_, allowing its isolation.

In part 2, we confirm that [**2**]^2–^ allows the reconstitution of apo-HydA1 to give active enzyme containing Se in place of S in the [2Fe]_H_ subunit. The differing efficiencies of maturation with serine vs CH_2_O as the carbon source provides unique insights into the adt-forming stage in the biosynthesis. This difference implicates a role for S–S bond formation with the lipoate cofactor that is not compatible with Se. This last piece of the puzzle presumably involves HydF and multiple components of the glycine cleavage system.

Hydrogen evolution assays demonstrate that *Cr*HydA1-Se_2_ is highly active. EPR and ENDOR spectroscopic analyses indicate that the [2Se]-H-cluster and the native H-cluster possess distinct, but largely similar geometric and electronic structures, as well as comparable redox properties. More detailed characterization on the catalytic bias and hyperfine couplings may reveal subtler differences in *Cr*HydA1-Se_2_.

## Experimental Section

Materials and methods have been described recently.^37^ Unless noted otherwise, procedures were conducted under N_2_ at room temperature (RT) on stirred solutions. Samples were dried under vacuum prior to recording yields. Fe_2_(μ-Se_2_)­(CO)_6_ was prepared according to the literature.^58^ Chromatography was conducted on a 3 cm × 25 cm column of silica gel.

^1^H, ^13^C­{^1^H}, ^77^Se­{^1^H}, and ^1^H–^77^Se HSQC NMR spectra were recorded on Varian UNITY INOVA 500 MHz, Varian Inova 600 MHz, and Bruker Ascend 600 MHz spectrometers. Spectra were processed using MestReNova software package. Chemical shifts are reported using the δ scale (ppm) relative to SiMe_4_, using ^1^H and ^13^C residual chemical shifts of the solvent as a secondary standard. Coupling constants (*J*) are reported in Hz. Data were collected at room temperature (RT) unless otherwise indicated.

### [K­(18-crown-6)]_2_[Fe_2_(μ-Se_2_)­(CN)_2_(CO)_4_] ([K­(18-crown-6)]_2_[1])

In a drybox, a 20 mL vial was charged with Fe_2_(μ-Se_2_)­(CO)_6_ (302 mg, 0.690 mmol, 1.0 equiv) and 7 mL of THF. After cooling to −35 °C, the solution was treated with 18-crown-6 (383 mg, 1.45 mmol, 2.10 equiv), followed by KN­(SiMe_3_)_2_ (289 mg, 1.45 mmol, 2.10 equiv). The dark brown solution was allowed to warm to RT and stirred for 1 d, during which the product precipitated. The resulting suspension was allowed to settle, and the brown supernatant was removed by decantation. The resulting dark red microcrystals were washed with THF (6 × 2 mL) and Et_2_O (1 × 2 mL). Yield: 420 mg (58%). ^1^H NMR (CD_3_CN): δ 3.58 (s, C*H*_2_O from 18-crown-6). ^13^C­{^1^H} NMR (151 MHz, CD_3_CN): δ 222.95 (*C*O), 146.73 (*C*N), 70.95 (*C* from 18-crown-6). ^77^Se NMR (114 MHz, CD_3_CN): δ −271. FT-IR (MeCN, *v*/cm^–1^): 2078 (m, CN), 1971 (s, CO), 1920 (s, CO), 1895 (s, CO). ESI-MS: Calcd for [M + H]^−^: 436.7; found: 436.9. Anal. Calcd for C_30_H_48_Fe_2_K_2_N_2_O_16_Se_2_·C_4_H_8_O: C, 36.70; H, 5.07; N, 2.52; Found: C, 36.69; H, 5.17; N, 2.66. Single crystals suitable for crystallographic analysis were grown by layering a MeCN solution of [K­(18-crown-6)]_2_[**1**] with THF and Et_2_O at −35 °C.

### [K­(18-crown-6)]_2_[HFe_2_(μ-SeH)­(μ-Se)­(CN)_2_(CO)_4_] ([K­(18-crown-6)]_2_[2])

In a drybox, [K­(18-crown-6)]_2_[**1**] (150 mg, 0.144 mmol, 1.0 equiv) and 1,1′-bis­(trimethylsilyl)-4,4′-bipyridinylidene (70 mg, 0.231 mmol, 1.6 equiv) were loaded in a 20 mL vial. Cold (−35 °C) MeCN (4 mL) was added, followed by MeOH (92 mg, 2.88 mmol, 20.0 equiv). The reaction was allowed to warm to RT and stirred for a further 3 h. Solvent was removed. The dark brown residue was extracted into a small volume of MeCN (∼2 mL). This mixture was filtered through Celite. Atop this filtrate were layered THF (∼0.5 mL) and Et_2_O (∼2.5 mL). This mixture was allowed to stand for 48 h to allow crystallization of [K­(18-crown-6)]_2_[**2**]. After the supernatant had been decanted, the resulting dark brown solid was washed with THF (4 × 1 mL). Yield: 111 mg (74%). FT-IR (MeCN, ν/cm^–1^): 2092 (m, CN), 2001 (s, CO), 1979 (s, CO), 1933 (s, CO). Anal. Calcd for C_30_H_50_Fe_2_K_2_N_2_O_16_Se_2_·C_4_H_8_O: C, 34.56; H, 4.83; N, 2.69; Found: C, 34.32; H, 4.88; N, 3.06.

### TEMPO Oxidation of [K­(18-crown-6)]_2_[2]

In a drybox, [K­(18-crown-6)]_2_[**2**] (50 mg, 0.048 mmol) and TEMPO (23 mg, 0.144 mmol) were placed in a 20 mL vial. Approximately 3 mL of Et_2_O was added, and the resulting suspension was stirred at room temperature for 48 h. The dark red solid was allowed to settle, and the supernatant was decanted. After washing with Et_2_O, the residue was identified as [K_2_(18-crown-6)_2_]­[**1**] on the basis of its FT-IR spectrum. Yield: 46 mg (92%).

The synthesis of [Fe_2_[(μ-^77^SeCH_2_)_2_NH]­(CN)_2_(CO)_4_]^2–^ ([^77^ **3**]^2–^) followed our previously published procedure,^31^ with the modification that natural abundance Se powder was replaced by ^77^Se.

#### Fe_2_[(μ-^77^SeCH_2_)_2_NCbz]­(CO)_6_

^1^H NMR (C_6_D_6_): δ 7.16–7.04 (m, 5H, Ar*H*), 5.09 (br, 2H, PhC*H*_2_), 4.22–2.56 (br, 4H, 2 × SeC*H*_2_). ^13^C­{^1^H} NMR (151 MHz, CD_3_CN): δ 208.03, 151.93, 135.63, 68.64, 36.91, 36.33, 35.76. ^77^Se­{^1^H} NMR (114 MHz, C_6_D_6_): δ 111.74 (d, *J*_Se–Se_ = 47.2 Hz), 108.61 (d, *J*_Se–Se_ = 47.1 Hz). ^77^Se NMR (114 MHz, C_6_D_6_): δ 111.76 (dt, *J*_Se–Se_ = 47.2, *J*_Se–H_ = 19.8 Hz), 108.62 (dt, *J*_Se–Se_ = 47.1, *J*_Se–H_ = 20.0 Hz). FT-IR (CH_2_Cl_2_, *v*/cm^–1^): 2070 (m, CO), 2032 (s, CO), 1994 (s, CO), 1715 (m, C = O). ESI-MS: calcd for [M + H]^+^, 611.8; found: 612.0.

#### Fe_2_[(μ-^77^SeCH_2_)_2_NH]­(CO)_6_

^1^H NMR (C_6_D_6_): δ 2.86–2.72 (m, 4H, 2 × SeC*H*_2_), 0.94 (m, 1H, N*H*). ^13^C­{^1^H} NMR (151 MHz, CD_3_CN): δ 209.76, 208.32, 41.06, 40.92, 40.73, 40.63, 40.43, 40.30. ^77^Se­{^1^H} NMR (114 MHz, C_6_D_6_): δ 7.27 (s). ^77^Se NMR (114 MHz, C_6_D_6_): δ 7.25 (m). FT-IR (CH_2_Cl_2_, *v*/cm^–1^): 2065 (m, CO), 2026 (s, CO), 1990 (s, CO). ESI-MS: calcd for [M + H]^+^, 477.7; found: 478.0.

#### (NEt_4_)_2_[Fe_2_[(μ-^77^SeCH_2_)_2_NH]­(CN)_2_(CO)_4_] ((NEt_4_)_2_[^77^3])

^1^H NMR (CD_3_CN): δ 3.53 (br, 2H, SeC*H*_2_), 3.37 (br, 2H, SeC*H*_2_), 3.18 (br, 16H, 8 × C*H*_2_CH_3_), 2.39 (b, 1H, N*H*), 1.21 (br, 24H, 8 × CH_2_C*H*_3_). ^13^C­{^1^H} NMR (151 MHz, CD_3_CN): δ 221.43, 220.06, 151.96, 150.51, 53.68, 38.39, 38.26, 37.89, 37.76, 37.72, 8.33. FT-IR (MeCN, *v*/cm^–1^): 2073 (m, CN), 1959 (s, CO), 1918 (s, CO), 1882 (s, CO). ESI-MS: calcd for [M + H]^−^, 473.7; found: 473.9.

### Defined Maturation of CrHydA1

Maturation of *Cr*HydA1 follows previously reported procedures.^31^ The maturation reaction used *Chlamydomonas reinhardtii* HydA1 containing the [4Fe-4S]_H_ cluster (apo-*Cr*HydA1), *Shewanella oneidensis* HydF (*So*HydF), *E. coli* serine hydroxymethyltransferase (*Ec*SHMT) and *E. coli* aminomethyltransferase (glycine cleavage system T-protein, *Ec*AMT) and necessary small molecules.

Purification of these proteins followed the same reported protocol. Briefly, apo-*Cr*HydA1 containing an N-terminal Strep-II tag placed in a pET-21­(b) plasmid was overexpressed in an *E. coli* BL21­(DE3) Δ*iscR:kan* strain, and purified using a Strep-tag affinity chromatography in a gravity column (Strep-Tactin resin, IBA). Excess desthiobiotin was removed by PD-10 G-25 desalting column (Cytiva). SoHydF containing C-terminal 6xHis tag in pET-21b plasmid was also overexpressed in the *E. coli* BL21­(DE3) Δ*iscR:kan* strain, and purified using the cobalt affinity chromatography in a gravity column (HisPur Cobalt Resin, Thermo Fisher) according to the manufacturer’s manual. Excess imidazole was removed using the PD-10 G-25 desalting column. The gene constructs to express C-terminal 6xHis-tagged *Ec*SHMT (pET-28a-*Ec*SHMT-6xHis) and *Ec*AMT (pET-23a-*Ec*AMT-6xHis) were synthesized by GenScript. Both genes are from *E. coli* K-12 strain and the sequences are adapted from NCBI without any modification (accession # BAA16459.1 for SHMT and AAC75943.1 for AMT). Both proteins were overexpressed in *E. coli* BL21­(DE3) strain aerobically, purified using the cobalt affinity chromatography and desalted similarly. *Ec*SHMT is copurified with the pyridoxal phosphate (PLP) cofactor and shows a bright-yellowish color. *Ec*AMT is copurified with the tetrahydrofolate (THF) cofactor and shows a pale-yellowish color.

For the maturation of *Cr*HydA1 using [**2**]^2–^ and serine as the carbon source, a typical reaction (∼2 mL final volume in 50 mM HEPES 150 mM KCl pH 8.0 buffer) cocktail contained 0.5 mL of 200 μM *Cr*HydA1, 0.25 mL of 100 μM *So*HydF, 0.1 mL of 200 μM *Ec*SHMT, 0.1 mL of 100 μM *Ec*AMT, 0.5 mM pyridoxal phosphate (PLP), 0.5 mM tetrahydrofolate, 20 mM guanosine triphosphate (GTP), 1 mM dithionite, 4 mM dithiothreitol (DTT), 30 mM NH_4_Cl, 30 mM serine and 2 mM synthetic Fe compound. The maturation reaction was performed for 1 h in the Coy box under ∼2% H_2_/98% N_2_ atmosphere at room temperature. The reaction mixture was then centrifuged to remove any precipitate. The supernatant was first desalted to remove the remaining iron compounds and other small molecules. Maturated CrHydA1 was then repurified from the eluent using a small Strep-Tactin affinity column (∼5 mL resin) and concentrated to 300 μM for further characterizations.

For the maturation using CH_2_O as the carbon source, *Ec*SHMT and PLP was further omitted from the reaction mixture, and 30 mM CH_2_O (^13^C-labeled as necessary) was added to the reaction mixture to replace serine. This maturation reaction (∼2 mL final volume) contained a mixture of *Cr*HydA1, *So*HydF, *Ec*AMT, GTP, THF, dithionite, DTT, NH_4_Cl, CH_2_O and [**2**]^2–^.

For the reconstitution of CrHydA1 with [^77^ **3**]^2–^, *Cr*HydA1 was incubated with 20-fold excess [^77^ **3**]^2–^ for 20 min at room temperature. The resulted solution was desalted to remove excess small molecules and concentrated as desired for EPR and ENDOR characterization.

#### EPR Spectroscopy

EPR experiments were carried out in the CalEPR center at the University of California, Davis. X-band continuous-wave spectra were recorded on a Bruker BioSpin EleXsys E500 spectrometer equipped with a super high Q resonator (ER4122SHQE) at 15 K. Cryogenic temperature was achieved and controlled using an ESR900 liquid helium cryostat, a temperature controller (Oxford Instrument ITC503) and a gas flow controller. Q-band Mims electron–nuclear double resonance (ENDOR) experiments were performed on the Bruker BioSpin EleXsys E580 spectrometer equipped with a R. A. Isaacson cylindrical TE_011_ resonator.^60^ All measurements were performed at 15 K. Cryogenic temperatures were achieved and controlled with an Oxford Instrument CF935 cryostat. The following pulse sequences were used: electron spin echo-detected field sweep EPR (π/2-τ-π-τ-echo), Mims ENDOR (π/2-τ-π/2-RF-π/2-τ-echo), and Davies ENDOR (π-RF-π/2-τ-π-τ-echo). EPR spectral simulations were performed in Matlab 2025a (MathWorks, Inc.) using EasySpin 6.0.10 toolbox.^61^

EPR samples of thionine-oxidized *Cr*HydA1 were prepared in a N_2_-containing glovebox. To 50 μL of 300 μM *Cr*HydA1 was added thionine (not to be confused with the protein thionin) to a final concentration of 2 mM. The solution was mixed quickly and immediately transferred into the EPR sample tube and frozen in liquid nitrogen for further analysis.

#### Cryogenic Photolysis of HydA1

Due to the instability of the synthetic Fe­(CO)­(CN) compounds in aqueous solution and the release of CO, the maturated HydA1 and the H-cluster analogues contained substantial CO-inhibited form. As reported in the 1980s,^49,62^ H_ox_-CO undergoes photolysis to generate H_ox_ at low temperature (7–15 K), although the conversion was not complete. In our case, cryogenic photolysis of various HydA1 samples was performed on the X-band E500 spectrometer when the samples were loaded into the helium cryostat. A SCHOTT KL2500 Fiber Optic LED light source with an optic fiber was used to direct white LED to illuminate the EPR samples through the window on the resonator. Photolysis was performed using 100% light intensity for 20 min. CW EPR spectra were recorded before and after the photolysis. We found that the illuminated samples can be stored in liquid nitrogen for future use, but the released CO would rebind to the H-cluster if the samples were stored at −80 °C for prolonged time or illuminated at higher temperature (78 K).

#### H_2_ Production Assay of Maturated HydA1

H_2_ production assays were performed according to previous procedures.^35^ The reaction was setup in a glovebox with N_2_ atmosphere. A mixture of 0.1 μM CrHydA1 and 5 mM methyl viologen in 3 mL pH 6.8 phosphate buffer was sealed in a 15 mL tube. The reaction was initiated by injecting 30 μL freshly made 1 M sodium dithionite solution into the tube and was continued for ∼30 min with gentle shaking. H_2_ production was monitored every 10 min by injecting 500 μL headspace gas into a Varian 3800 gas chromatograph equipped with a 60/80 Å molecular sieve and the thermal conductive detector.

## Supplementary Material



## Notes

Spectroscopic data, selected reaction schemes, detailed procedures (PDF)

## §

Department of Chemistry, Tufts University, Medford, Massachusetts 02155, United States
