# Supplementary material for: Biosynthesis of the Selenium-Substituted [FeFe]-Hydrogenases
Source: J Am Chem Soc. 2026 Jun 16;148(25):26757–67. doi: 10.1021/jacs.6c08167 (PMC13339137; doi:10.1021/jacs.6c08167)
Supplement: Supplementary file 1 [file ja6c08167_si_001.pdf]

Supporting information for

Biosynthesis of the Selenium-Substituted [FeFe]-Hydrogenases

Xin Yu,[1] Toby Woods,[1] Yu Zhang,[1†] R. David Britt,[2*] Guodong Rao,[2*] and Thomas B. Rauchfuss[1*]

1. School of Chemical Sciences, University of Illinois, Urbana, IL 61801, USA
2. Department of Chemistry, University of California, Davis CA 95616, USA

Table of Contents

Figures 2S
Figures S1-S6. Spectra for [**1**]$^{2-}$ 2S-5S
Figures S7-15. Spectra for [**2**]$^{2-}$ 6S-10S
Figures S16-23. Spectra for Cbz derivatives 11S-14S
Figures S24-30. Spectra for ^{77}Se-(CO)$_6$ derivatives. 15S-18S
Figure S31-35. Spectra for ^{77}Se-(CN)$_2$(CO)$_4$ derivatives 19S-21S
Defined maturation of CrHydA1 .... 22S
X-ray crystal structure analysis 26S
References 27S

[†] Present address: Department of Chemistry, Tufts University, Medford, MA

Figures

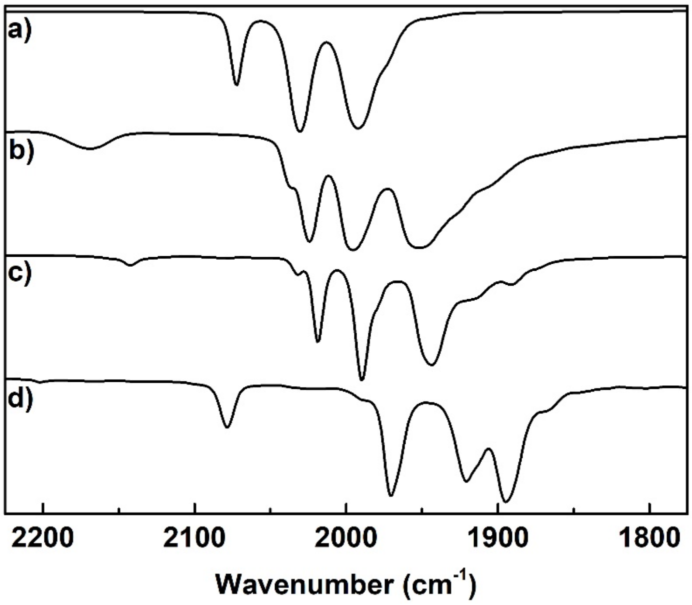

Figure S1. IR spectra of (a) $Fe_2(\mu\text{-}Se_2)(CO)_6$ in THF, (b) $Fe_2(\mu\text{-}Se_2)(CO)_6$ reaction with 2 equiv $KN(tms)_2$ in THF, c) "b" after added 2 equiv 18-crown-6 in THF and (d) $[K(18\text{-crown-6})]_2[Fe_2(\mu\text{-}Se_2)(CN)_2(CO)_4]$ ($[K(18\text{-crown-6})]_2$[**1**]) in MeCN.

Results:

a) v/cm^{-1}: 2072 (s, CO), 2031 (s, CO), 1992 (s, CO).

b) v/cm^{-1}: 2169 (m, CN), 2024 (s, CO), 1996 (s, CO), 1952 (s, CO).

c) v/cm^{-1}: 2142 (w, CN), 2019 (s, CO), 1990 (s, CO), 1944 (s, CO).

d) v/cm^{-1}: 2078 (m, CN), 1971 (s, CO), 1921 (s, CO), 1895 (s, CO).

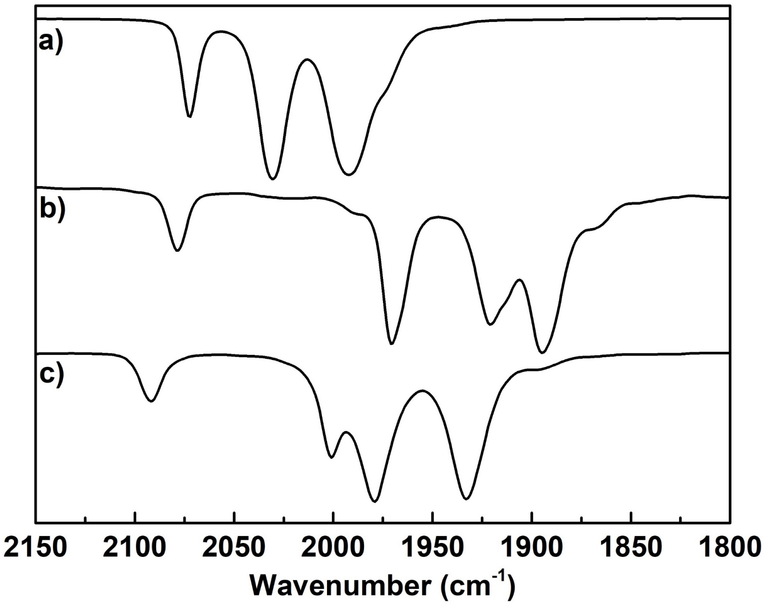

Figure S2. IR spectra of (a) $Fe_2(\mu\text{-}Se_2)(CO)_6$ in THF, (b) $[K(18\text{-crown-}6)]_2[Fe_2(\mu\text{-}Se_2)(CN)_2(CO)_4]$ ($[K(18\text{-crown-}6)]_2$[**1**]) in MeCN and (c) $[K(18\text{-crown-}6)]_2[HFe_2(\mu\text{-}SeH)(\mu\text{-}Se)(CN)_2(CO)_4]$ ($[K(18\text{-crown-}6)]_2$[**2**]) in MeCN.

Results:

a) v/cm^{-1}: 2072 (s, CO), 2031 (s, CO), 1992 (s, CO).

b) v/cm^{-1}: 2078 (m, CN), 1971 (s, CO), 1921 (s, CO), 1895 (s, CO).

c) v/cm^{-1}: 2092 (m, CN), 2001 (s, CO), 1979 (s, CO), 1933 (s, CO).

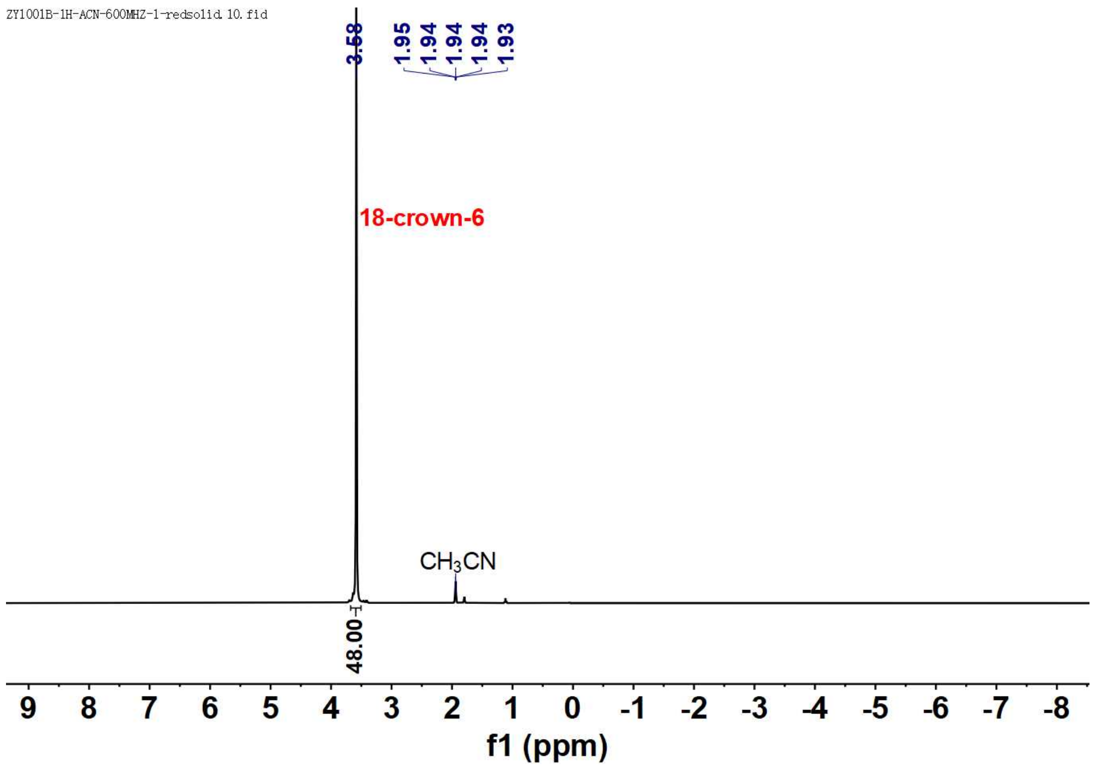

Figure S3. ^{1}H NMR spectrum of [K(18-crown-6)]$_2$[Fe_2(μ-Se_2)$(CN)_2$$(CO)_4$] ([K(18-crown-6)]$_2$[**1**]) in CD_3CN.

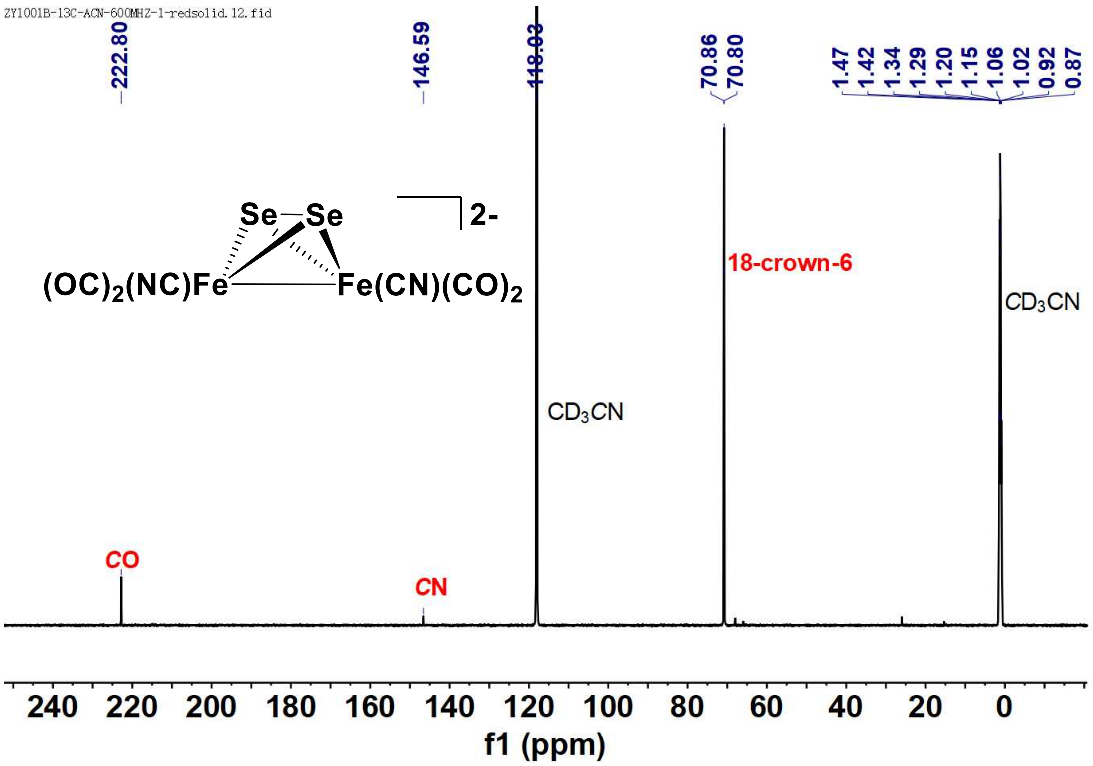

Figure S4. $^{13}C\{^{1}H\}$ NMR spectrum of [K(18-crown-6)]$_2$[Fe_2(μ-Se_2)$(CN)_2$$(CO)_4$] ([K(18-crown-6)]$_2$[**1**]) in CD_3CN.

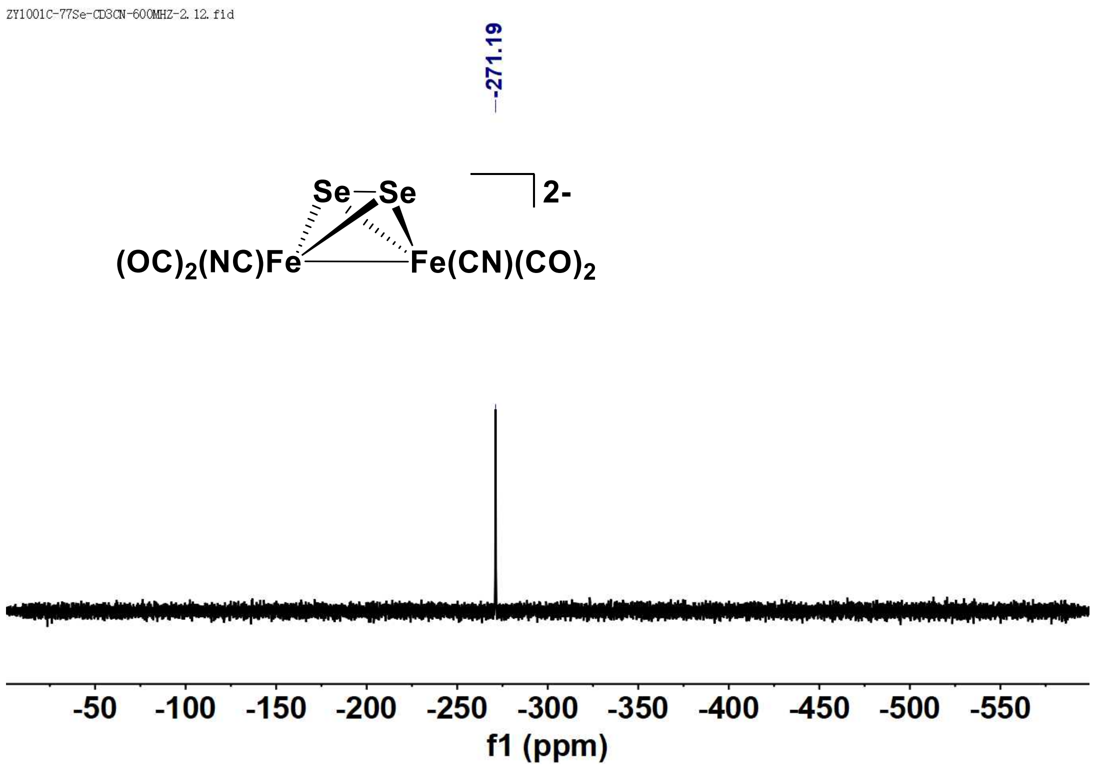

Figure S5. ^{77}Se NMR spectrum of [K(18-crown-6)]$_2$[Fe_2(μ-Se_2)$(CN)_2$$(CO)_4$] ([K(18-crown-6)]$_2$[**1**]) in CD_3CN.

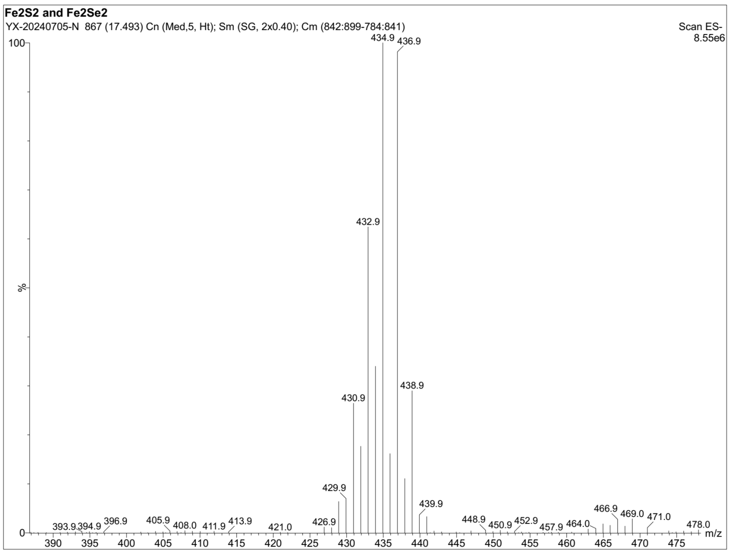

Figure S6. ESI-MS spectrum of [K(18-crown-6)]$_2$[Fe_2(μ-Se_2)$(CN)_2$$(CO)_4$] ([K(18-crown-6)]$_2$[**1**]).

Result: Calcd for $[M+H]^-$,436.7, found: 436.9.

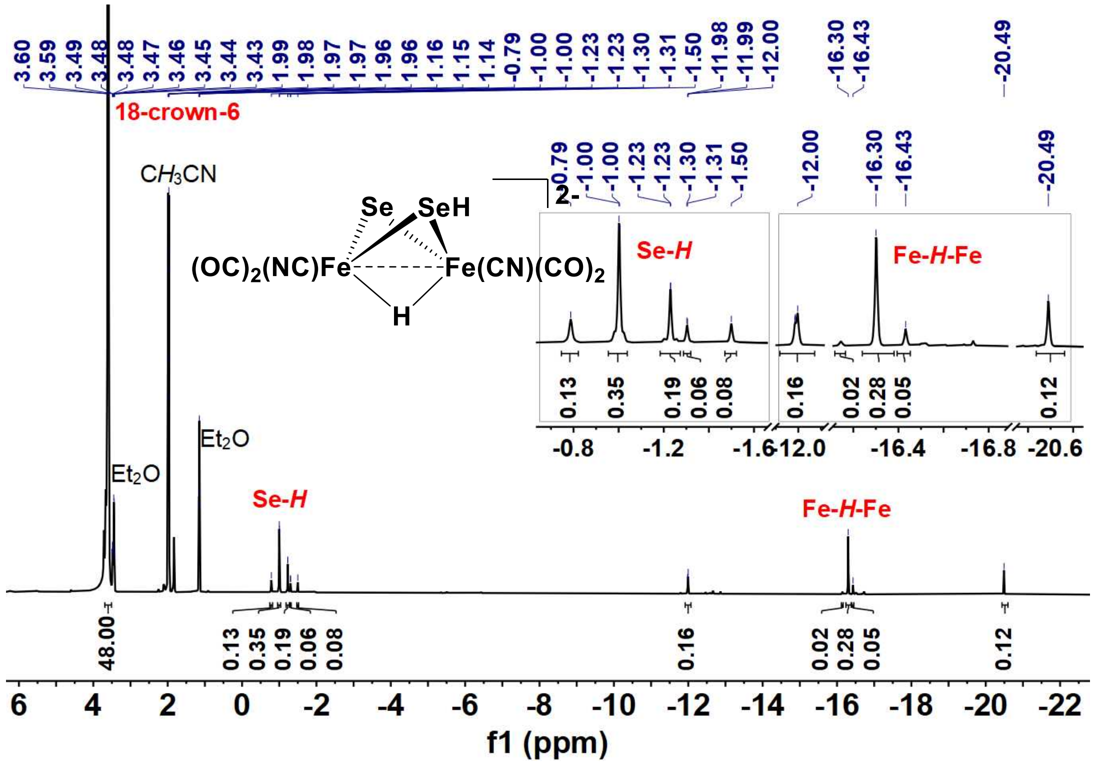

Figure S7. ^{1}H NMR spectrum of [K(18-crown-6)]$_2$[HFe$_2$(μ-SeH)(μ-Se)(CN)$_2$(CO)$_4$] ([K(18-crown-6)]$_2$[**2**]) in CD$_3$CN.

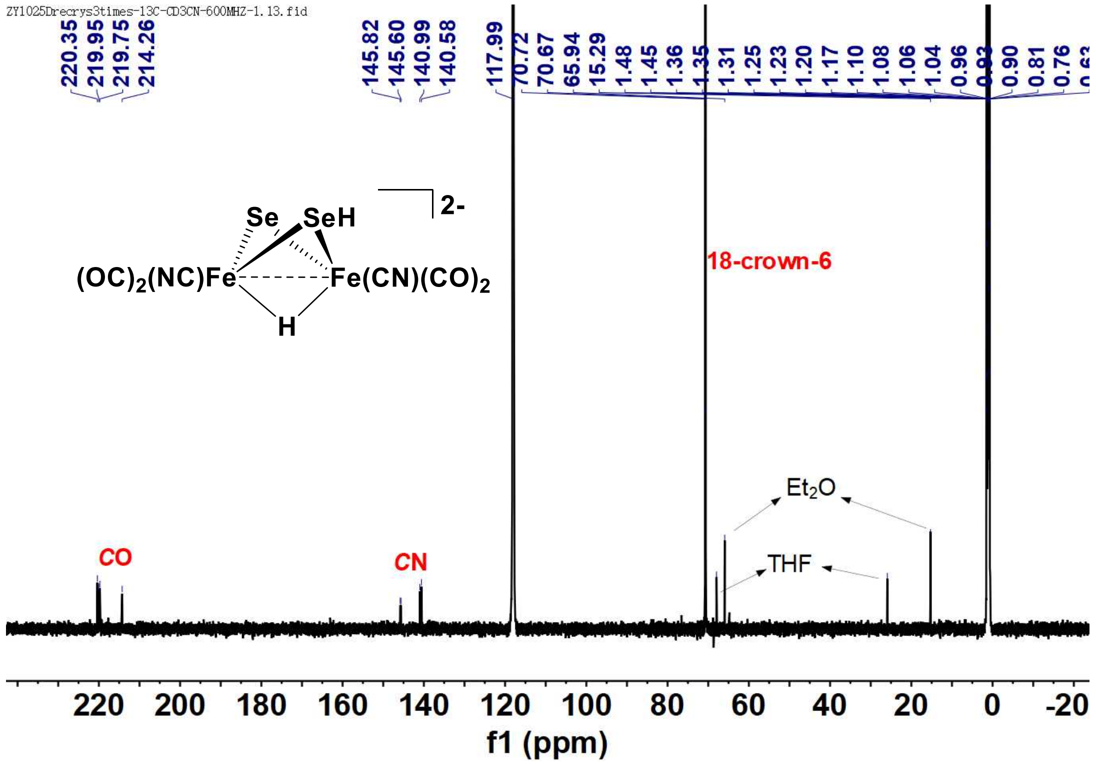

Figure S8. ^{13}C{^{1}H} NMR spectrum of [K(18-crown-6)]$_2$[HFe$_2$(μ-SeH)(μ-Se)(CN)$_2$(CO)$_4$] ([K(18-crown-6)]$_2$[**2**]) in CD$_3$CN.

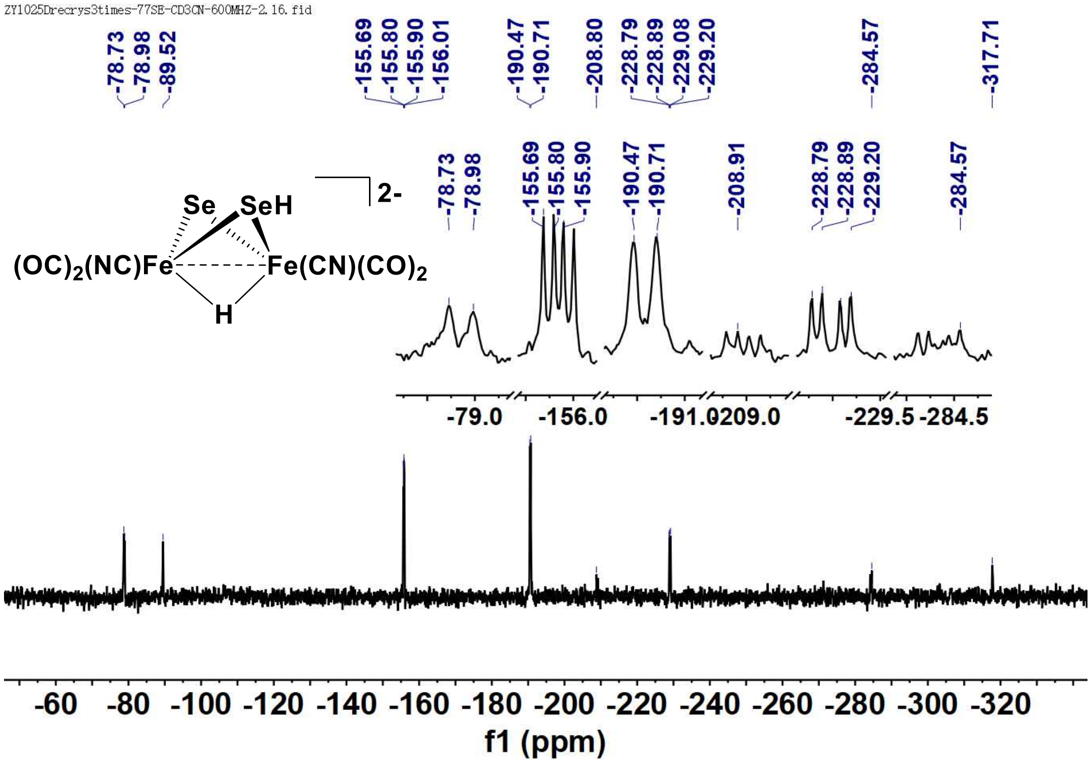

Figure S9. ^{77}Se NMR spectrum of [K(18-crown-6)]$_2$[HFe$_2$(μ-SeH)(μ-Se)(CN)$_2$(CO)$_4$] ([K(18-crown-6)]$_2$[**2**]) in CD_3CN.

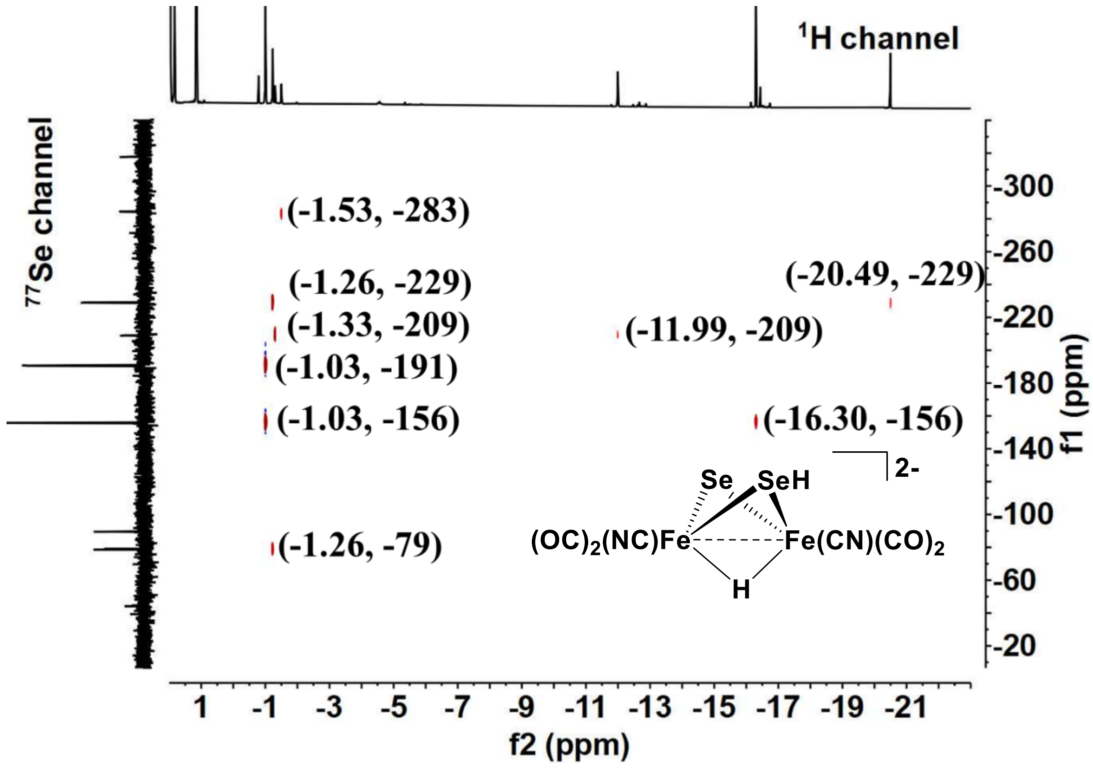

Figure S10. ^{1}H-^{77}Se HSQC spectrum of [K(18-crown-6)]$_2$[HFe$_2$(μ-SeH)(μ-Se)(CN)$_2$(CO)$_4$] ([K(18-crown-6)]$_2$[**2**]) in CD_3CN.

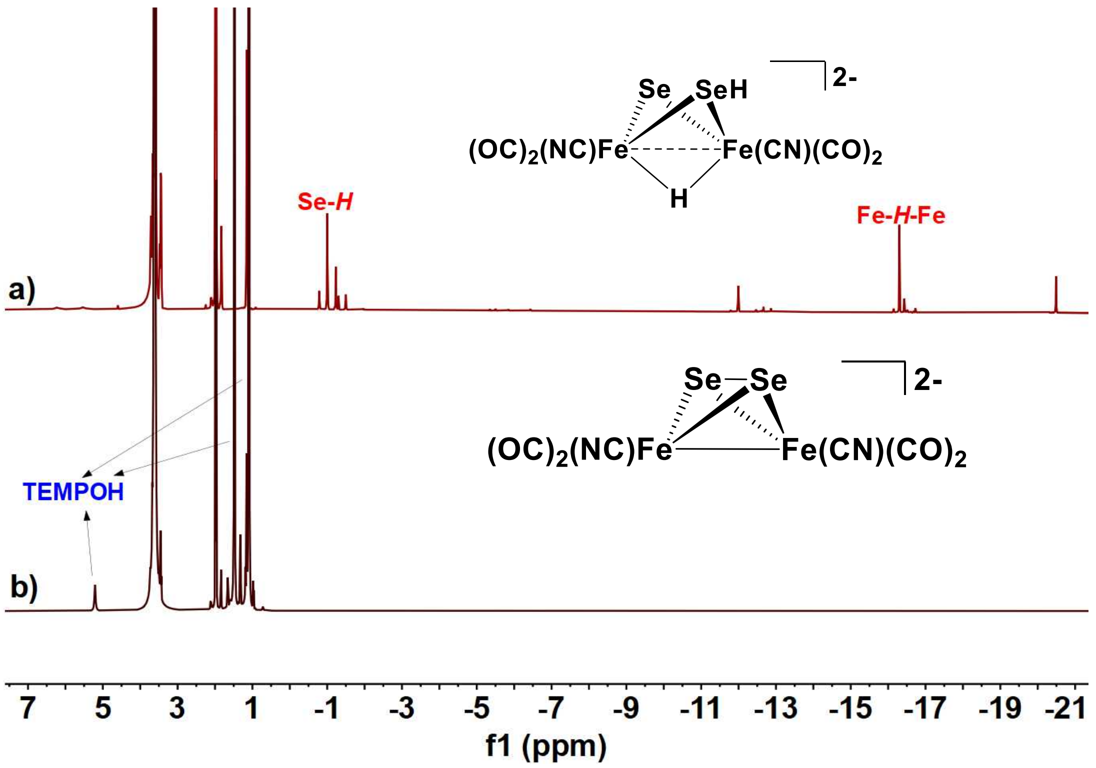

Figure S11. ^{1}H NMR spectra of (a) [K(18-crown-6)]$_2$[**2**] and (b) after addition of 2 equiv TEMPO in CD_3CN.

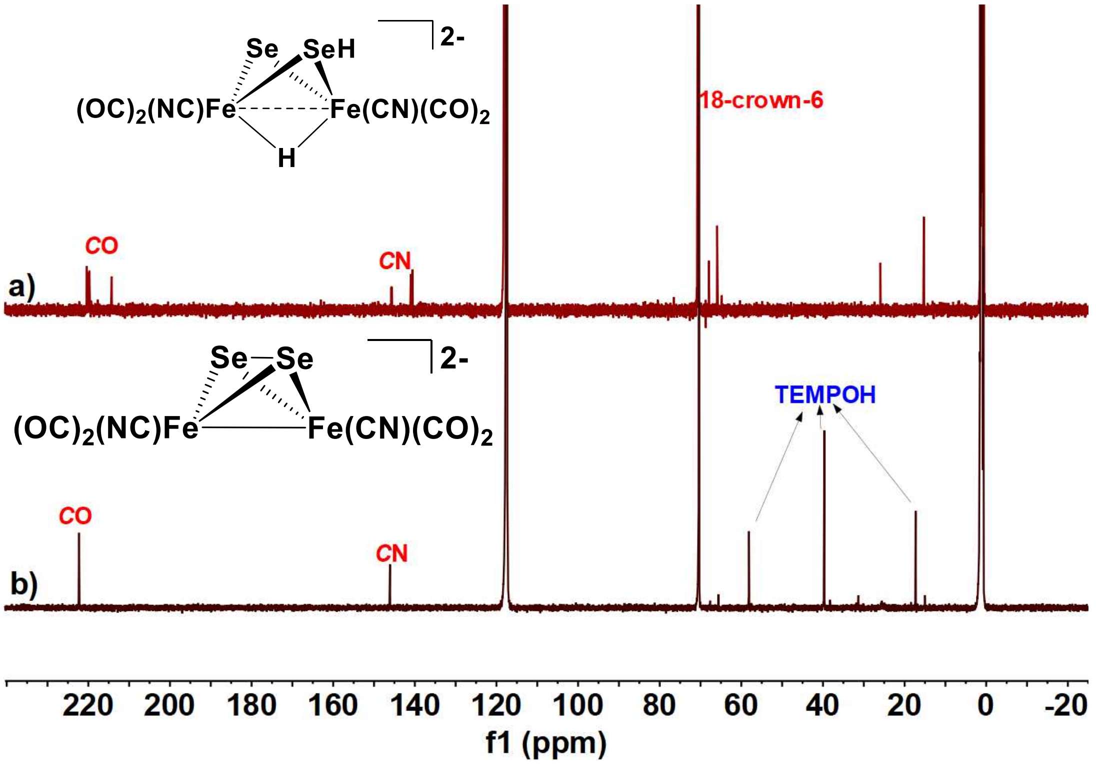

Figure S12. ^{13}C{^{1}H} NMR spectra of (a) [K(18-crown-6)]$_2$[**2**] and (b) after addition of 2 equiv TEMPO in CD_3CN.

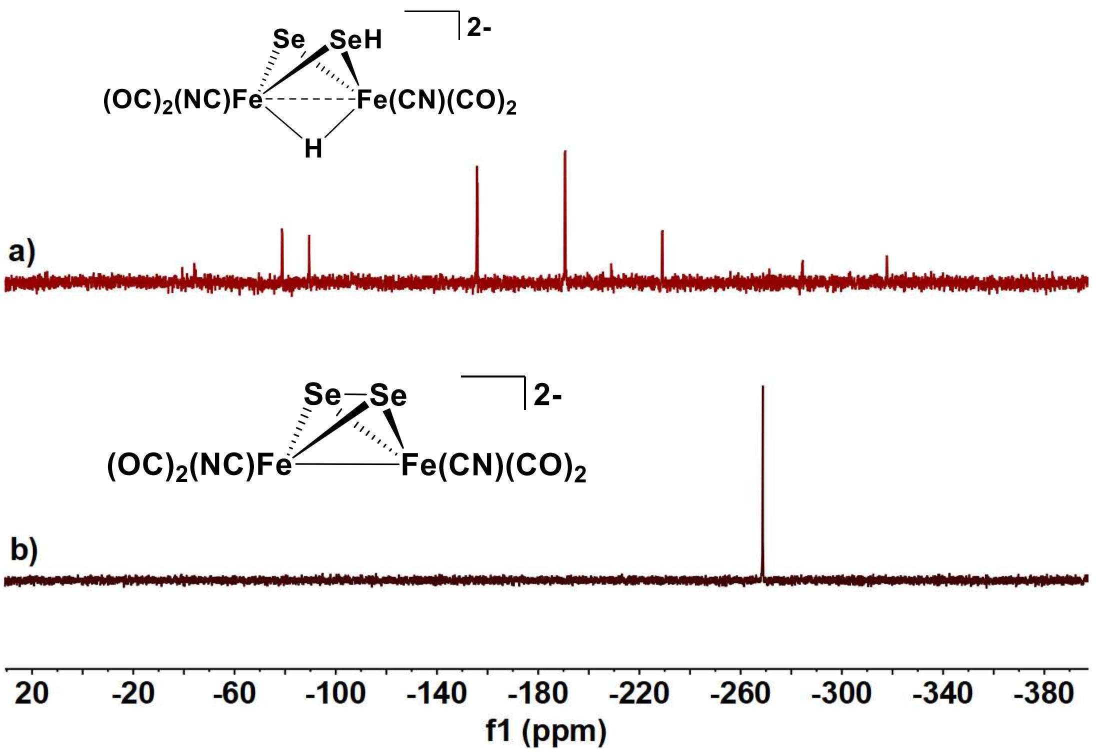

Figure S13. ^{77}Se NMR spectra of (a) [K(18-crown-6)]$_2$[**2**] and (b) after addition of 2 equiv TEMPO in CD_3CN.

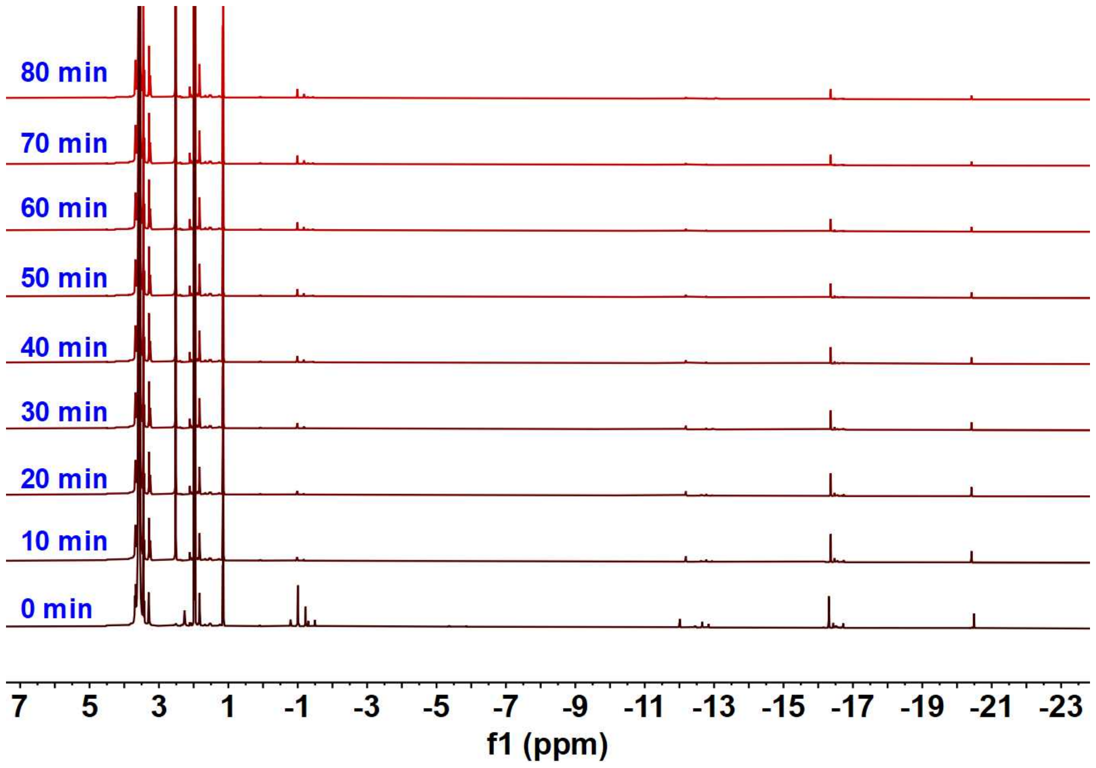

Figure S14. ^{1}H NMR spectra of the reaction between [K(18-crown-6)]$_2$[**2**] and 40 equiv CD_3OD in CD_3CN at various time.

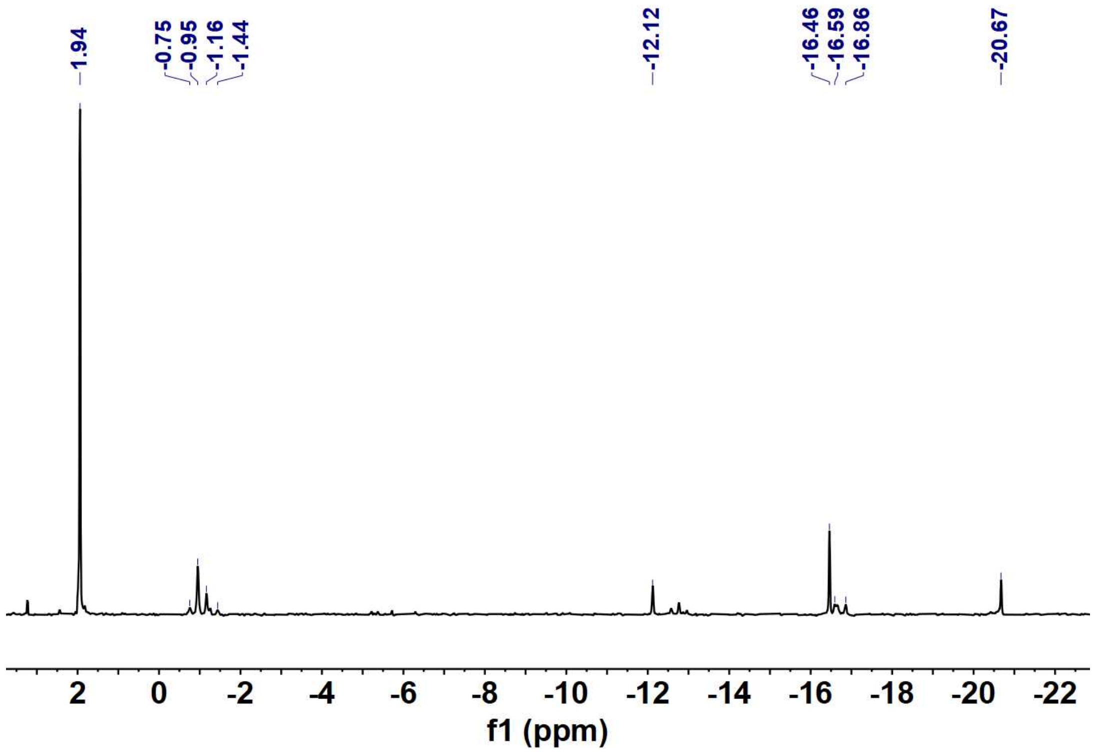

Figure S15. ^{2}H NMR spectrum of $[K(18\text{-crown-6})]_2[DFe_2(\mu\text{-SeD})(\mu\text{-Se})(CN)_2(CO)_4]$ in CH_3CN.

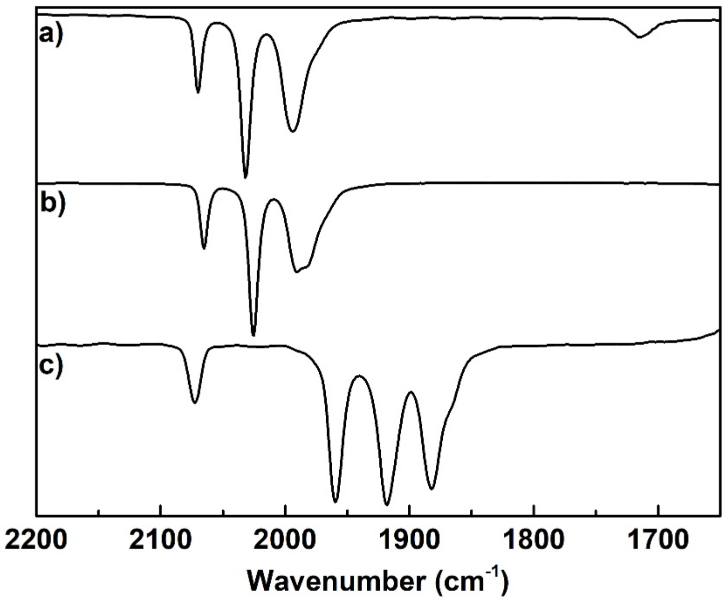

Figure S16. IR spectra of (a) $Fe_2[(\mu\text{-}^{77}SeCH_2)_2NCbz](CO)_6$ in CH_2Cl_2, (b) $Fe_2[(\mu\text{-}^{77}SeCH_2)_2NH](CO)_6$ in CH_2Cl_2, and (c) $(NEt_4)_2[Fe_2[(\mu\text{-}^{77}SeCH_2)_2NH](CN)_2(CO)_4]$ ($(NEt_4)_2[^{77}$**3**$]$) in MeCN.

Results:

a) v/cm^{-1}: 2070 (m, CO), 2032 (s, CO), 1994 (s, CO), 1715 (m, C=O).

b) v/cm^{-1}: 2065 (m, CO), 2026 (s, CO), 1990 (s, CO).

c) v/cm^{-1}: 2073 (m, CN), 1960 (s, CO), 1918 (s, CO), 1882 (s, CO).

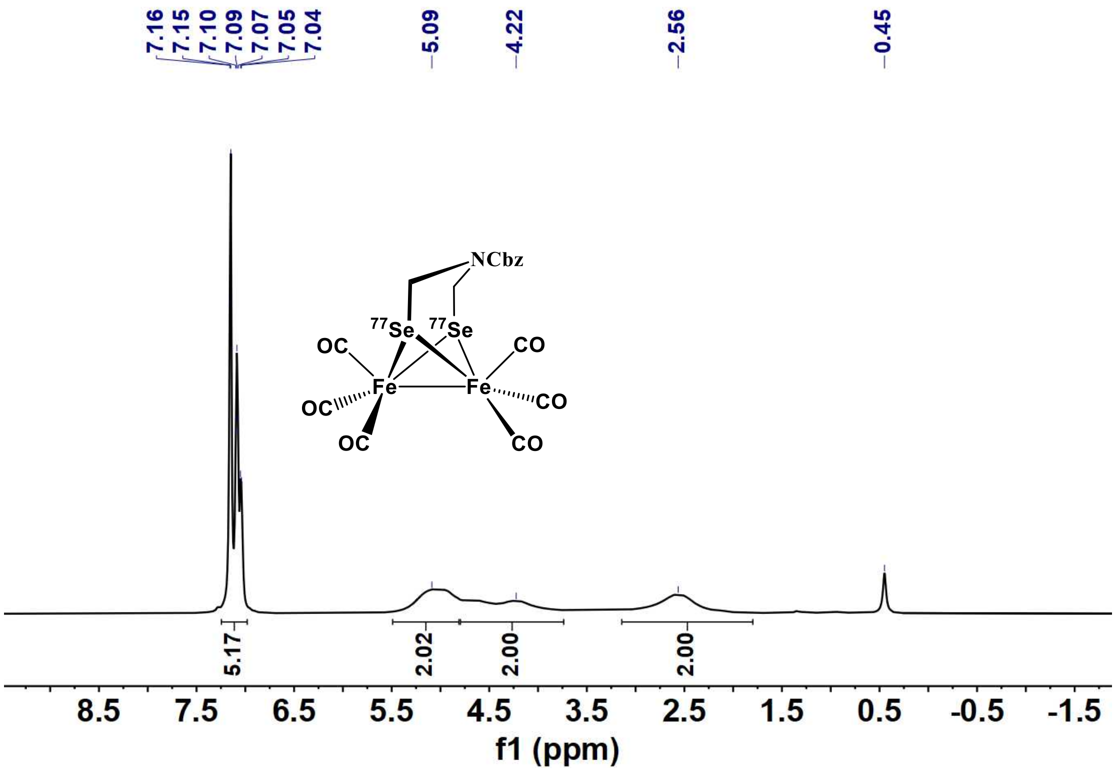

Figure S17. ^{1}H NMR spectrum of $Fe_2[(\mu\text{-}^{77}SeCH_2)_2NCbz](CO)_6$ in C_6D_6.

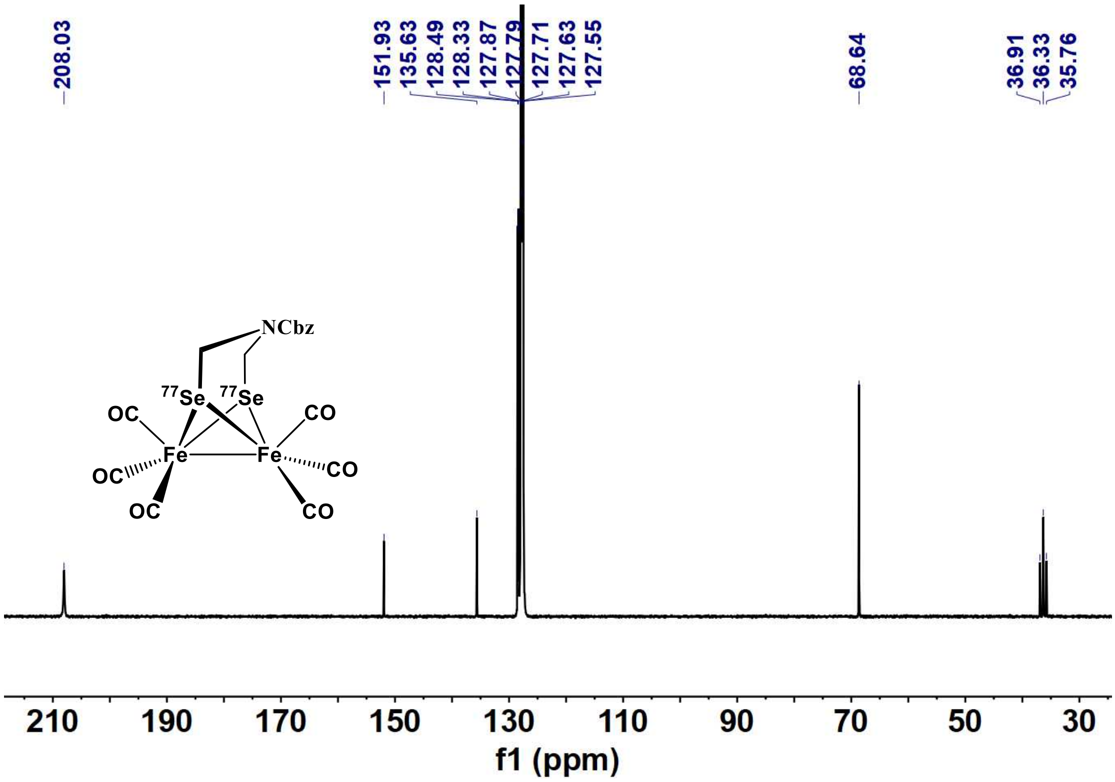

Figure S18. $^{13}C\{^{1}H\}$ NMR spectrum of $Fe_2[(\mu\text{-}^{77}SeCH_2)_2NCbz](CO)_6$ in C_6D_6.

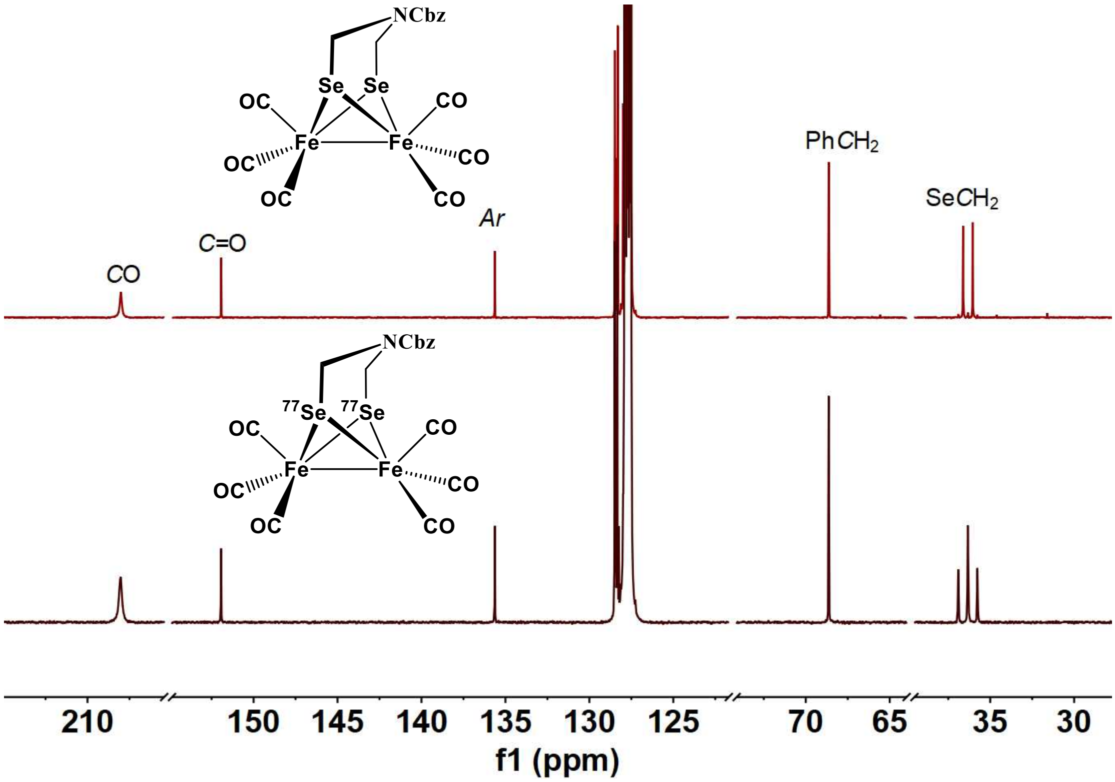

Figure S19. Comparison of ${}^{13}C\{{}^{1}H\}$ NMR spectra of $Fe_2[(\mu\text{-}SeCH_2)_2NCbz](CO)_6$ (top) and $Fe_2[(\mu\text{-}{}^{77}SeCH_2)_2NCbz](CO)_6$ (bottom) in C_6D_6.

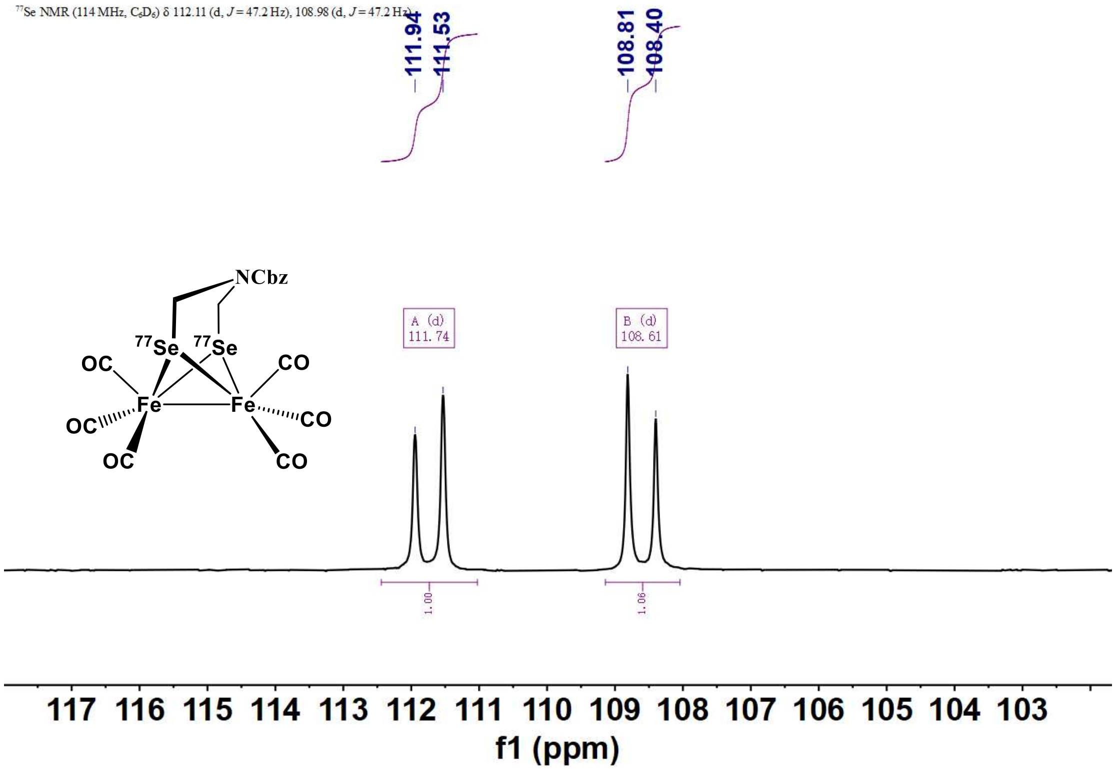

Figure S20. ${}^{77}Se\{{}^{1}H\}$ NMR spectrum of $Fe_2[(\mu\text{-}{}^{77}SeCH_2)_2NCbz](CO)_6$ in C_6D_6.

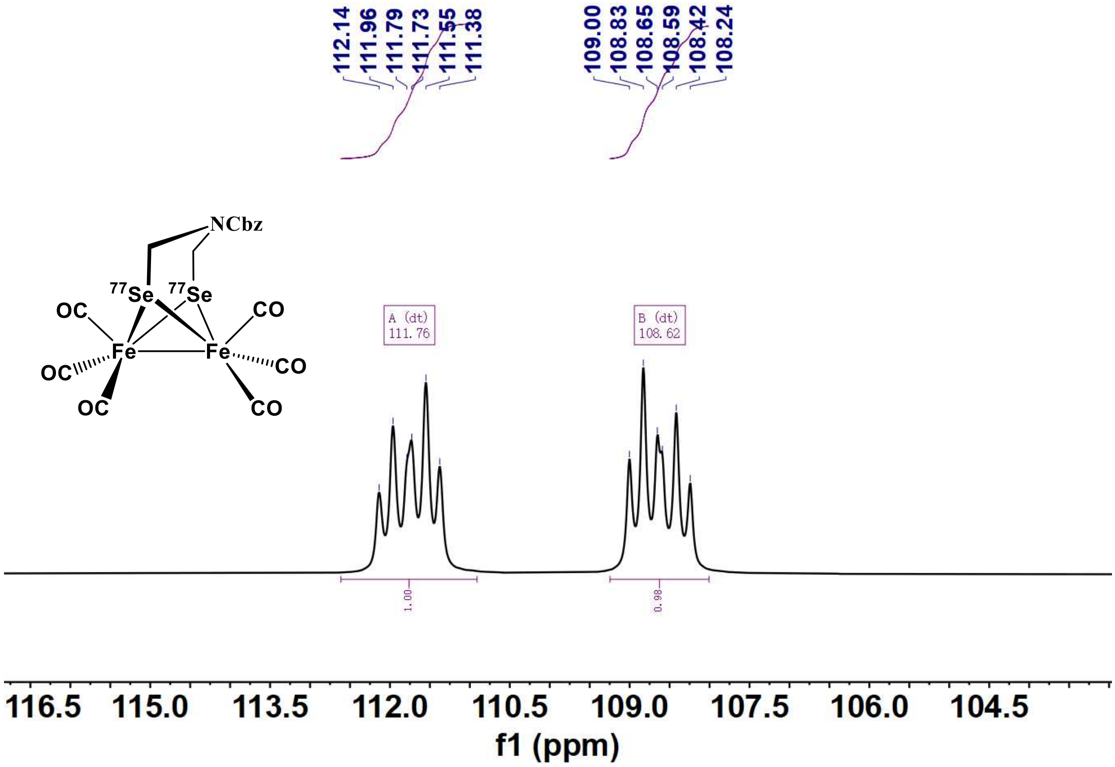

Figure S21. ^{77}Se NMR spectrum of $Fe_2[(\mu\text{-}^{77}SeCH_2)_2NCbz](CO)_6$ in C_6D_6.

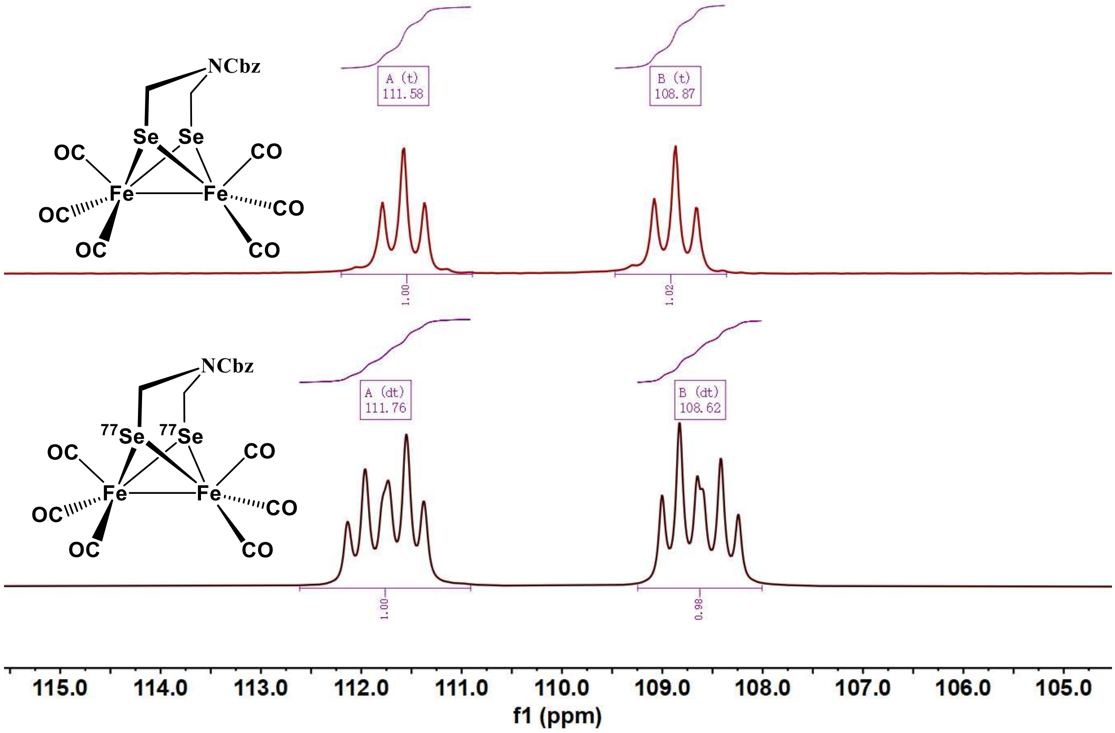

Figure S22. Comparison of ^{77}Se NMR spectra of $Fe_2[(\mu\text{-}SeCH_2)_2NCbz](CO)_6$ (top) and $Fe_2[(\mu\text{-}^{77}SeCH_2)_2NCbz](CO)_6$ (bottom) in C_6D_6.

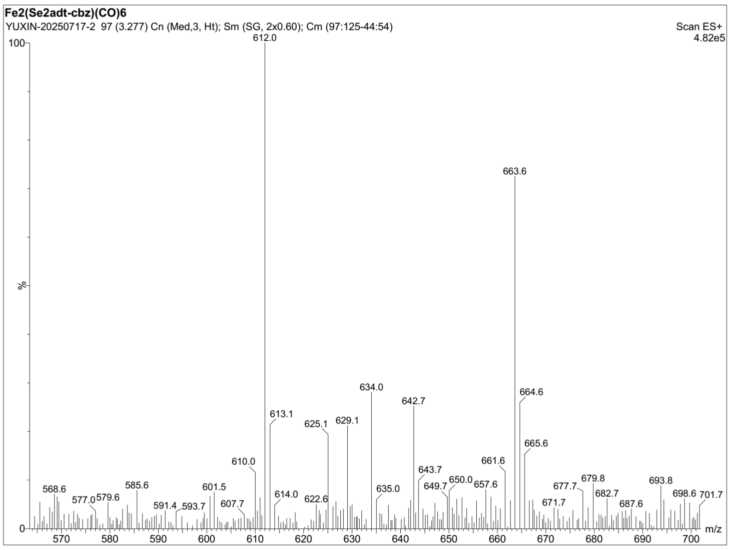

Figure S23. ESI-MS spectrum of $Fe_2[(\mu\text{-}^{77}SeCH_2)_2NCbz](CO)_6$.

Result: Calcd for $[M+H]^+$, 611.8; found: 612.0.

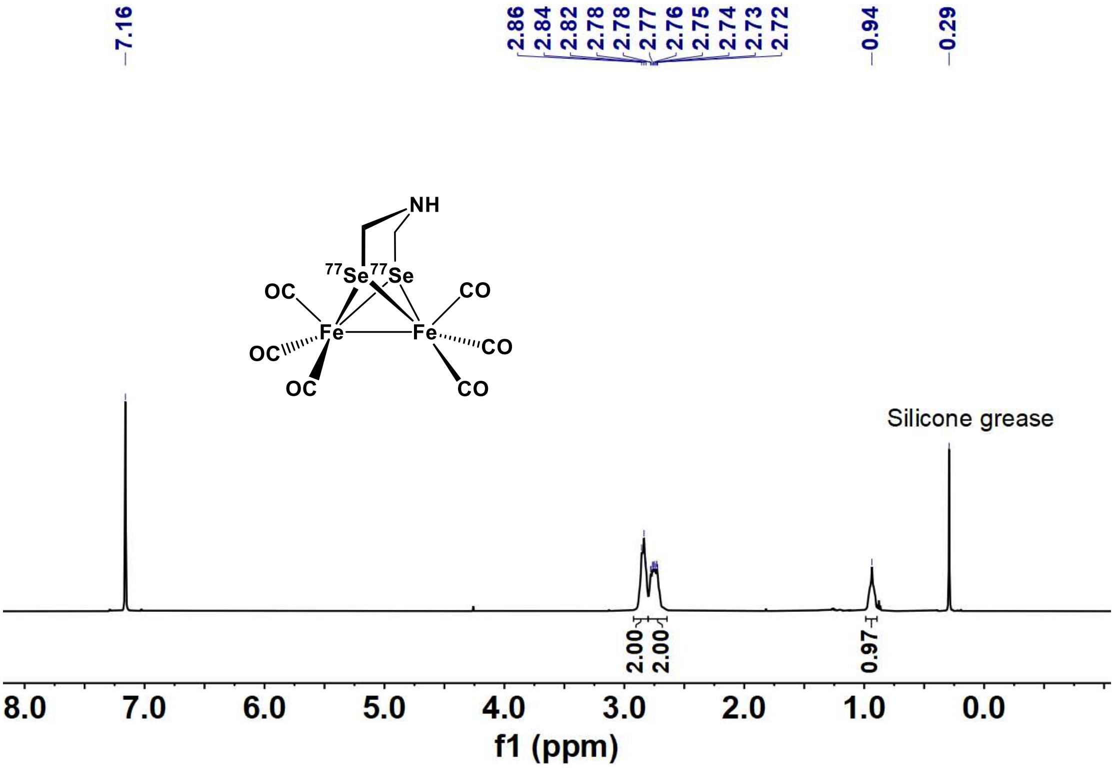

Figure S24. ^1H NMR spectrum of $Fe_2[(\mu\text{-}^{77}SeCH_2)_2NH](CO)_6$ in C_6D_6.

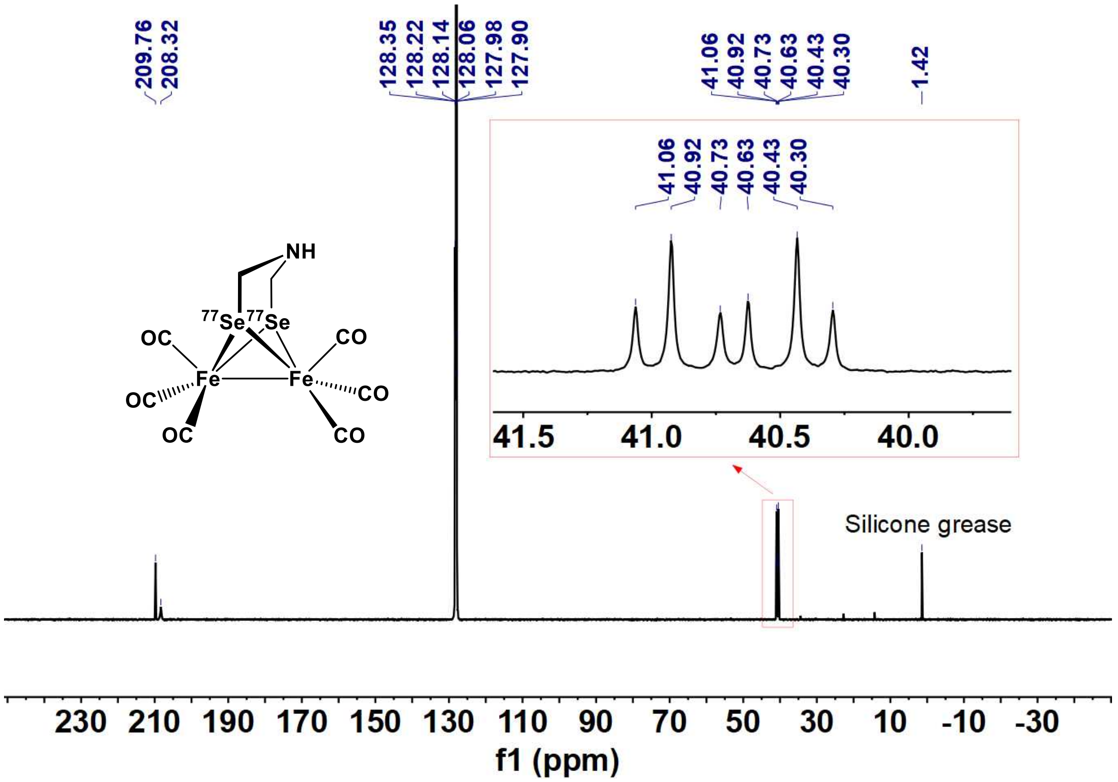

Figure S25. $^{13}C\{^{1}H\}$ NMR spectrum of $Fe_2[(\mu\text{-}^{77}SeCH_2)_2NH](CO)_6$ in C_6D_6.

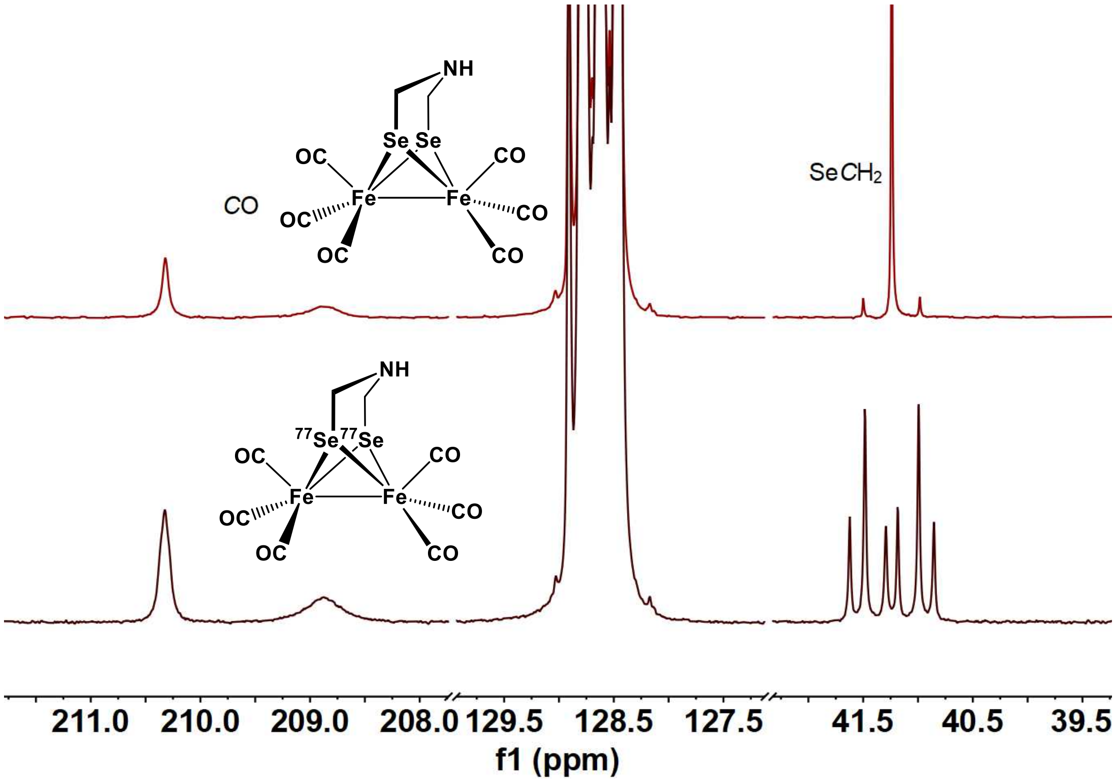

Figure S26. Comparison of $^{13}C\{^{1}H\}$ NMR spectra of $Fe_2[(\mu\text{-}SeCH_2)_2NH](CO)_6$ (top) and $Fe_2[(\mu\text{-}^{77}SeCH_2)_2NH](CO)_6$ (bottom) in C_6D_6.

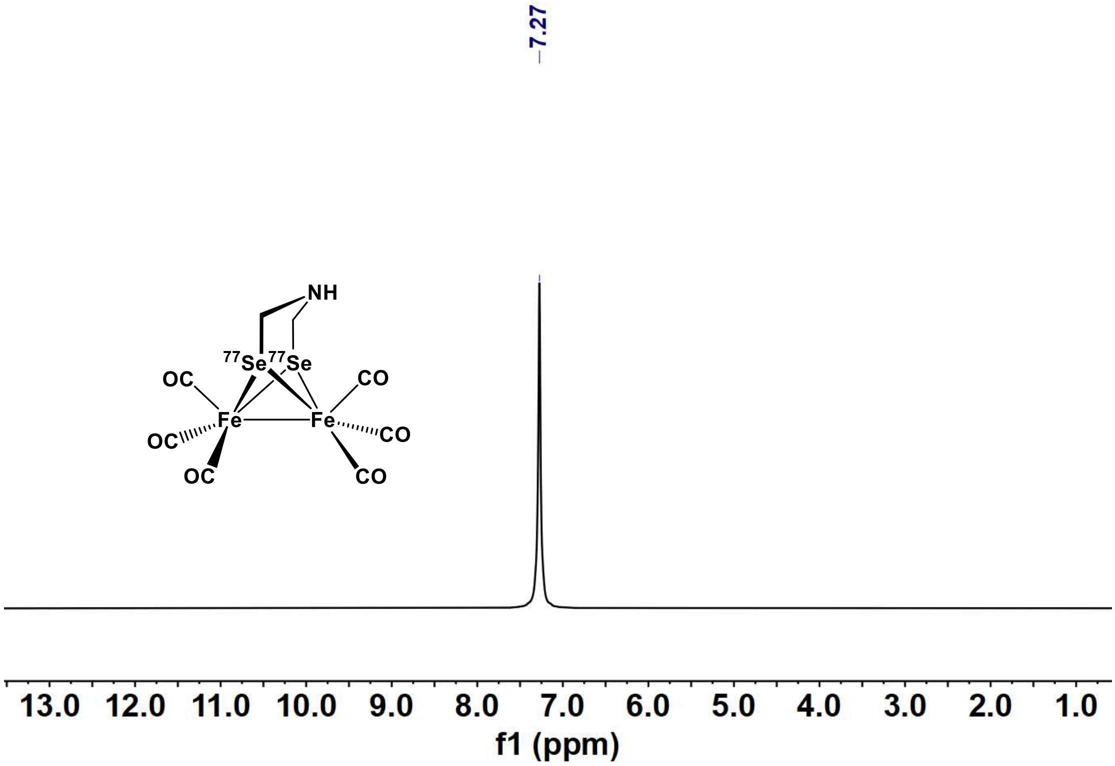

Figure S27. $^{77}Se\{^{1}H\}$ NMR spectrum of $Fe_2[(\mu\text{-}^{77}SeCH_2)_2NH](CO)_6$ in C_6D_6.

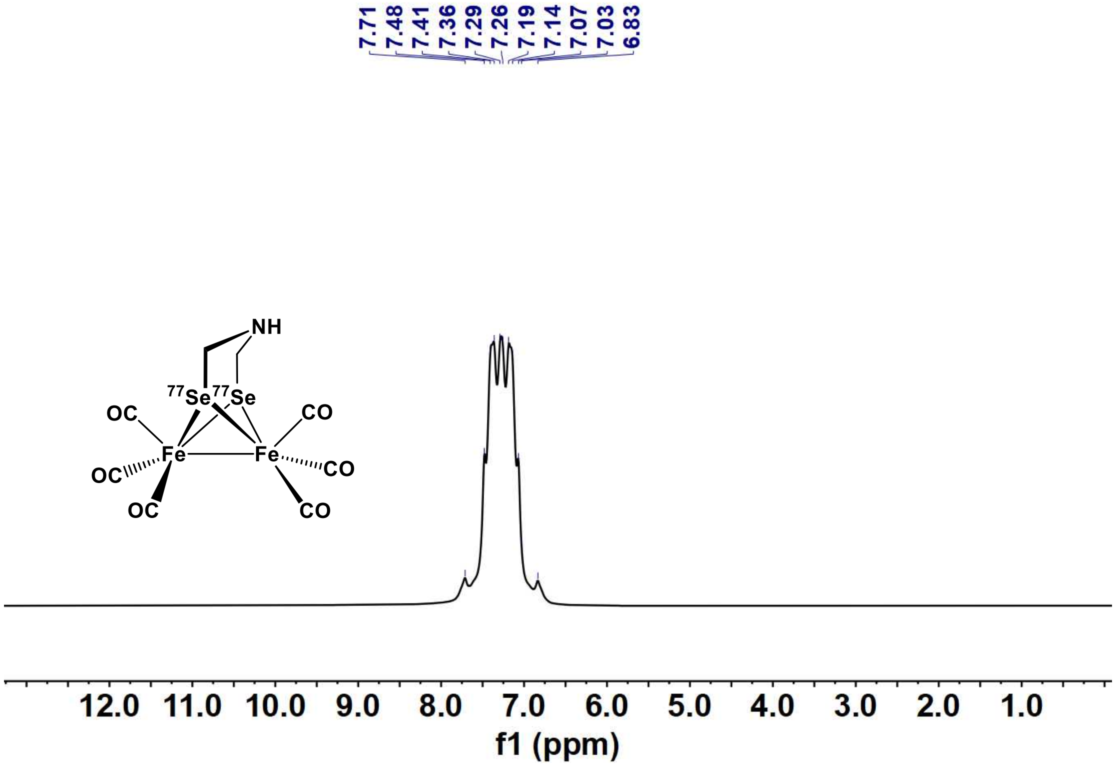

Figure S28. ^{77}Se NMR spectrum of $Fe_2[(\mu\text{-}^{77}SeCH_2)_2NH](CO)_6$ in C_6D_6.

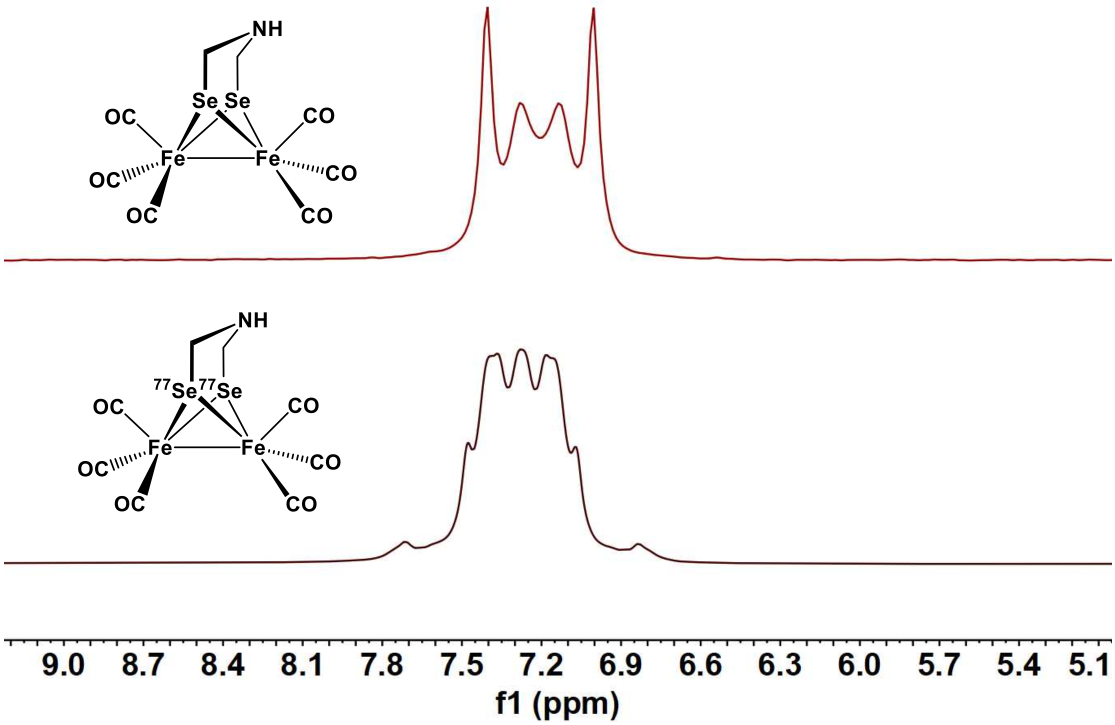

Figure S29. Comparison of ^{77}Se NMR spectra of $Fe_2[(\mu\text{-}SeCH_2)_2NH](CO)_6$ (top) and $Fe_2[(\mu\text{-}^{77}SeCH_2)_2NH](CO)_6$ (bottom) in C_6D_6.

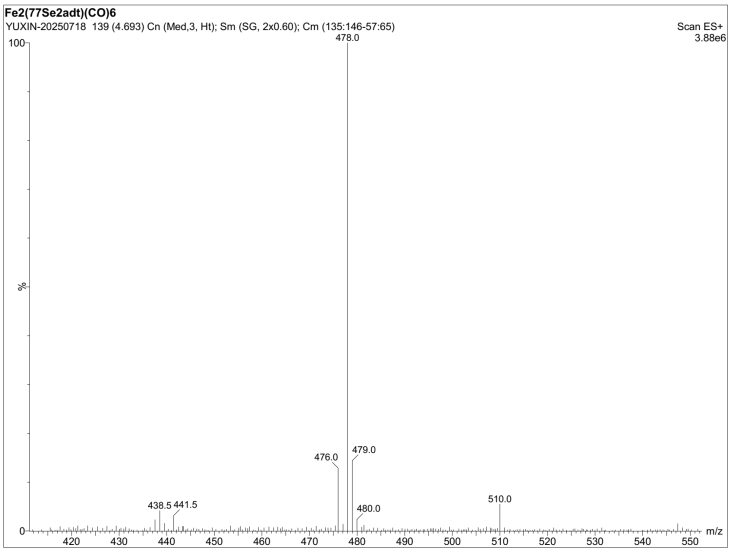

Figure S30. ESI-MS spectrum of $Fe_2[(\mu\text{-}^{77}SeCH_2)_2NH](CO)_6$.

Result: Calcd for $[M+H]^+$, 477.7; found:478.0.

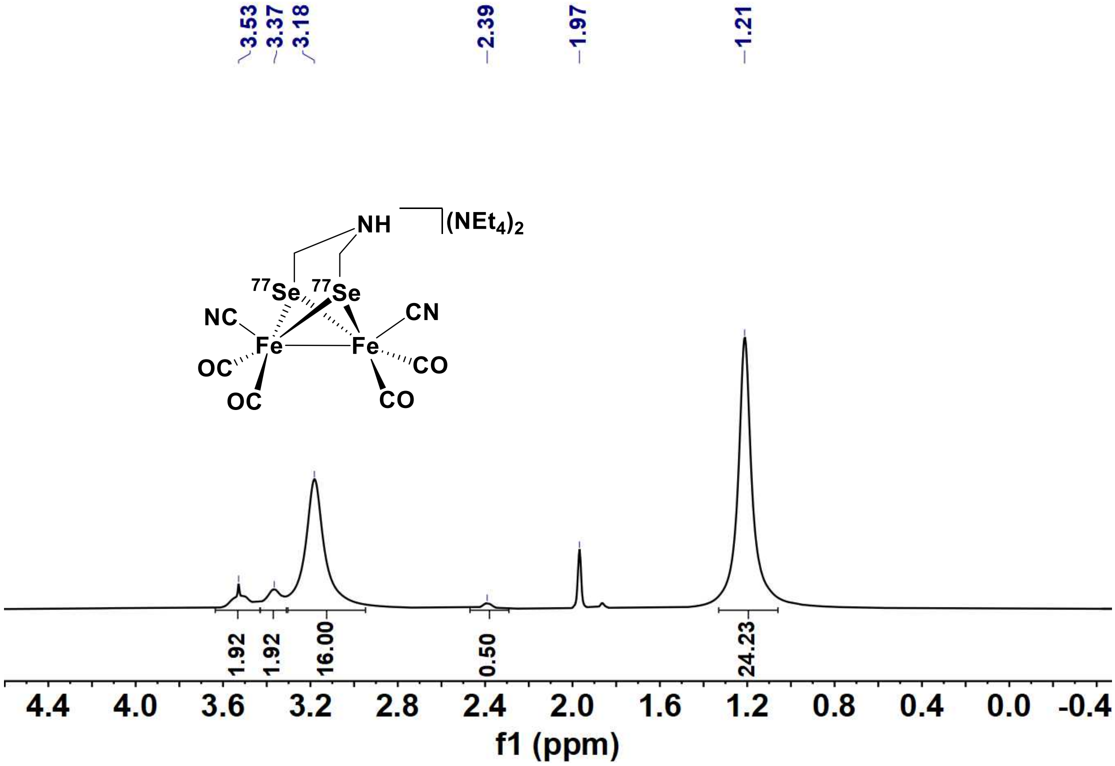

Figure S31. ^{1}H NMR spectrum of $(NEt_4)_2[Fe_2[(\mu\text{-}^{77}SeCH_2)_2NH](CN)_2(CO)_4]$ $(NEt_4)_2[^{77}\mathbf{3}]$ in CD_3CN.

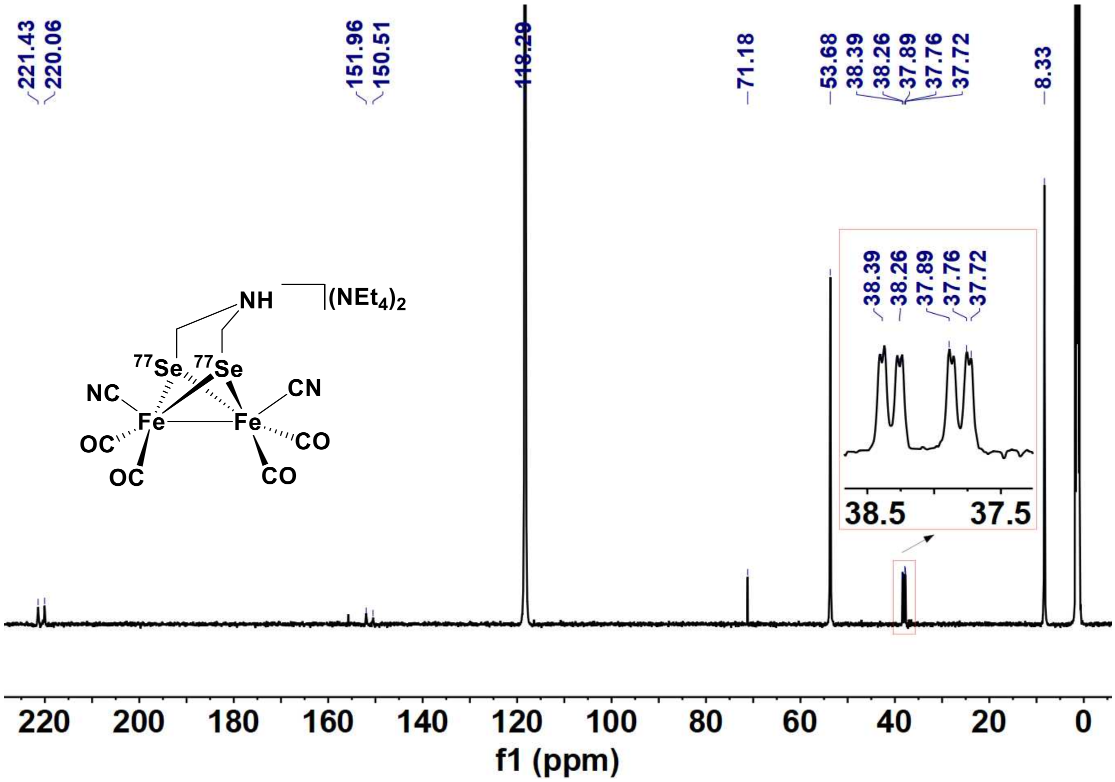

Figure S32. $^{13}C\{^{1}H\}$ NMR spectrum of $(NEt_4)_2[Fe_2[(\mu\text{-}^{77}SeCH_2)_2NH](CN)_2(CO)_4]$ $(NEt_4)_2[^{77}\mathbf{3}]$ in CD_3CN.

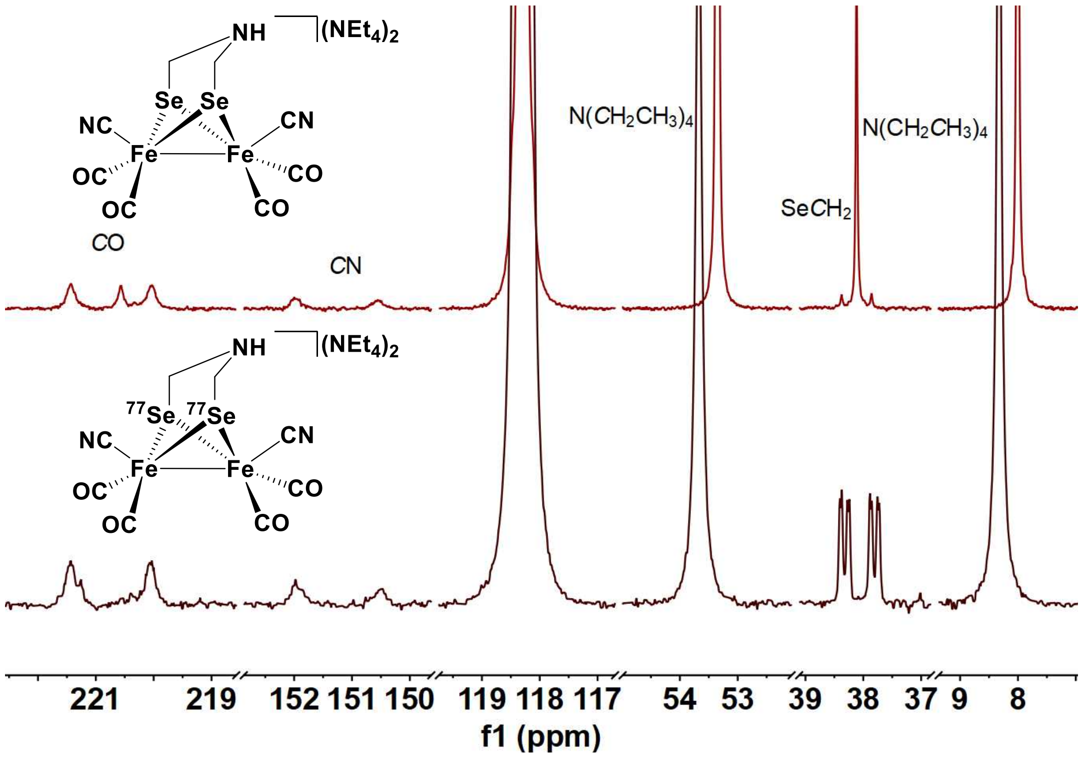

Figure S33. Comparison of $^{13}C\{^1H\}$ NMR spectra of $(NEt_4)_2[Fe_2[(\mu\text{-}SeCH_2)_2NH](CN)_2(CO)_4]$ $(NEt_4)_2$[**3**] (top) and $(NEt_4)_2[Fe_2[(\mu\text{-}{}^{77}SeCH_2)_2NH](CN)_2(CO)_4]$ $(NEt_4)_2[^{77}$**3**] (bottom) in CD_3CN.

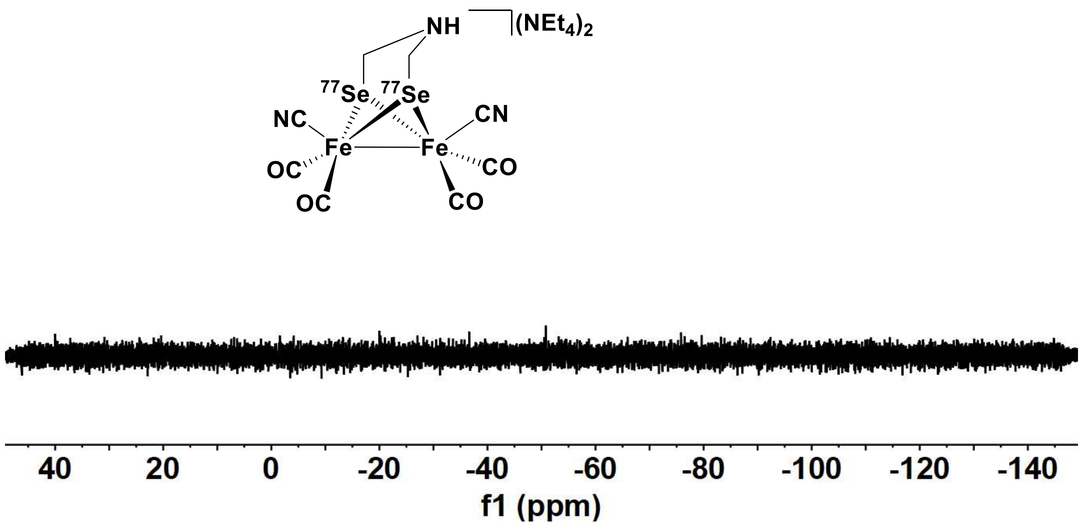

Figure S34. $^{77}Se\{^1H\}$ NMR spectrum of $(NEt_4)_2[Fe_2[(\mu\text{-}{}^{77}SeCH_2)_2NH](CN)_2(CO)_4]$ $(NEt_4)_2[^{77}$**3**] in CD_3CN at room temperature.

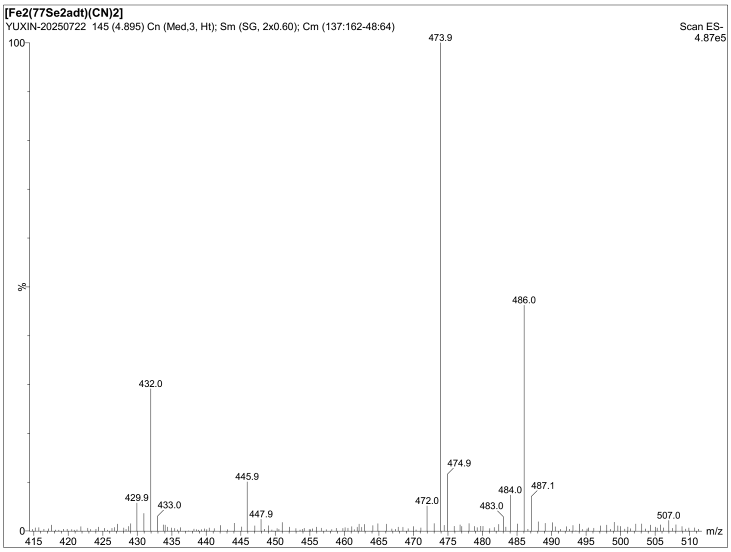

Figure S35. ESI-MS spectrum of $(NEt_4)_2[^{77}\mathbf{3}]$.

Result: Calcd for $[M+H]^-$, 473.7; found: 473.9.

Defined maturation of CrHydA1

Maturation of CrHydA1 follows previously reported procedures.[1] The maturation reaction used *Chlamydomonas reinhardtii* HydA1 containing the $[4Fe\text{-}4S]_H$ cluster (apo-*Cr*HydA1), S*hewanella oneidensis* HydF (*So*HydF), *E. coli* serine hydroxymethyltransferase (*Ec*SHMT) and *E. coli* aminomethyltransferase (glycine cleavage system T-protein, *Ec*AMT) and necessary small molecules.

Purification of these proteins followed the same reported protocol. Briefly, apo-*Cr*HydA1 containing an N-terminal Strep-II tag placed in a pET-21(b) plasmid was overexpressed in an *E. coli* BL21(DE3) *ΔiscR::kan* strain, and purified using a Strep-tag affinity chromatography in a gravity column (Strep-Tactin® resin, IBA). Excess desthiobiotin was removed by PD-10 G-25 desalting column (Cytiva). SoHydF containing C-terminal 6xHis tag in pET-21b plasmid were also overexpressed in the *E. coli* BL21(DE3) *ΔiscR::kan* strain, and purified using the cobalt affinity chromatography in a gravity column (HisPur™ Cobalt Resin, Thermo Fisher) according to the manufacturer's manual. Excess imidazole was removed using the PD-10 G-25 desalting column. The gene constructs to express C-terminal 6xHis-tagged *Ec*SHMT (pET-28a-*Ec*SHMT-6xHis) and *Ec*AMT (pET-23a-*Ec*AMT-6xHis) were synthesized by GenScript™. Both genes are from *E. coli* K-12 strain and the sequences are adapted from NCBI without any modification (accession # BAA16459.1 for SHMT and AAC75943.1 for AMT). Both proteins were overexpressed in *E. coli* BL21(DE3) strain aerobically, purified using the cobalt affinity chromatography and desalted similarly. *Ec*SHMT is co-purified with the pyridoxal phosphate (PLP) cofactor and shows a bright-yellowish color. *Ec*AMT is co-purified with the tetrahydrofolate (THF) cofactor and shows a pale-yellowish color.

For the maturation of *Cr*HydA1 using Fe_2Se_2 or $Fe_2(SeH)_2$ using serine as the carbon source, a typical reaction (~2 mL final volume in 50 mM HEPES 150 mM KCl pH 8.0 buffer) cocktail contained 0.5 mL of 200 μM *Cr*HydA1, 0.25

mL of 100 μM *So*HydF, 0.1 mL of 200 μM *Ec*SHMT, 0.1 mL of 100 μM *Ec*AMT, 0.5 mM pyridoxal phosphate (PLP), 0.5 mM tetrahydrofolate, 20 mM guanosine triphosphate (GTP), 1 mM dithionite, 4 mM dithiothreitol (DTT), 30 mM NH_4Cl, 30 mM serine and 2 mM synthetic compound. The maturation reaction was performed for 1 hrs in the Coy box under ~2% H_2/98% N_2 atmosphere at room temperature. The reaction mixture was then centrifuged to remove any precipitations. The supernatant was first desalted to remove the remaining iron compounds and other small molecules. Maturated CrHydA1 was then repurified from the eluent using a small Strep-Tactin affinity column (~5 mL resin) and concentrated to 300 μM for further characterizations.

For the maturation using CH_2O as the carbon source, *Ec*SHMT and PLP was further omitted from the reaction mixture, and 30 mM CH_2O (^{13}C-labeled as necessary) was added to the reaction mixture to replace serine. This maturation reaction (~2 mL final volume) contained a mixture of *Cr*HydA1, *So*HydF, *Ec*AMT, GTP, THF, dithionite, DTT, NH_4Cl, CH_2O and Fe_2Se_2 dimers.

For reconstituting CrHydA1 with $[^{77}\mathbf{3}]^{2-}$, *Cr*HydA1 was incubated with 20-fold excess $[^{77}\mathbf{3}]^{2-}$ for 20 min at room temperature. The resulted solution was desalted to remove excess small molecules and concentrated as desired for EPR and ENDOR characterization.

EPR spectroscopy. EPR experiments were carried out in the CalEPR center in the University of California, Davis. X-band continuous-wave spectra were recorded on a Bruker BioSpin EleXsys E500 spectrometer equipped with a super high Q resonator (ER4122SHQE) at 15 K. Cryogenic temperature was achieved and controlled using an ESR900 liquid helium cryostat, a temperature controller (Oxford Instrument ITC503) and a gas flow controller. Q-band Mims electron-nuclear double resonance (ENDOR) experiments were performed on the Bruker BioSpin EleXsys E580 spectrometer equipped with a a R.A. Isaacson cylindrical TE_{011} resonator.[2] All measurements were performed at 15 K. Cryogenic temperatures were achieved and controlled with an Oxford Instrument CF935 cryostat. The following pulse sequences were used: electron

spin echo-detected field sweep EPR (π/2-τ-π-τ-echo), Mims ENDOR (π/2-τ-π/2-RF-π/2-τ-echo), Davies ENDOR (π-RF-π/2-τ-π-τ-echo). EPR spectral simulations were performed in Matlab 2025a (MathWorks, Inc) using EasySpin 6.0.10 toolbox.[3]

EPR samples of thionine-oxidized *Cr*HydA1 were prepared in a N_2-containing glovebox. To 50 μL of 300 μM *Cr*HydA1 was added thionine (not to be confused with thionin, which is a protein) to a final concentration of 2 mM. The solution was mixed quickly and immediately transferred into the EPR sample tube and frozen in liquid nitrogen for further analysis.

Cryogenic photolysis of HydA1. Due to the instability of the synthetic Fe(CO)(CN) compounds in aqueous solution and the release of CO, the maturated HydA1 and the H-cluster analogues were primarily in the CO-inhibited form. As reported in the 1980s,[4-5] H_{ox}-CO undergoes photolysis to generate H_{ox} at low temperature (7-15 K), although the conversion was not complete. In our case, cryogenic photolysis of various HydA1 samples was performed on the X-band E500 spectrometer when the samples were loaded into the helium cryostat. A SCHOTT KL2500 Fiber Optic LED light source with an optic fiber was used to direct white LED to illuminate the EPR samples through the window on the resonator. Photolysis was performed using 100% light intensity for 20 min. CW EPR spectra were recorded before and after the photolysis. We found that the illuminated samples can be stored in the liquid nitrogen Dewar for future use, but the released CO would rebind to the H-cluster if the samples were stored at -80 °C for prolonged time or illuminated at higher temperature (78 K).

H_2 production assay of maturated HydA1. H_2 production assays were performed according to previous procedures.[6] The reaction was setup in a glovebox with N_2 atmosphere. A mixture of 0.1 μM CrHydA1 and 5 mM methyl viologen in 3 mL pH = 6.8 phosphate buffer was sealed in a 15 mL tube. The

reaction was initiated by injecting 30 μL freshly made 1 M sodium dithionite solution into the tube and was continued for ~ 30 min with gentle shaking. H_2 production was monitored every 10 min by injecting 500 μL headspace gas into a Varian 3800 gas chromatography equipped with a 60/80 Å molecular sieve and the thermal conductive detector.

X-ray crystal structure analysis

Table S1. Crystal data and structure refinement parameters for [K_2(18-crown-6)$_2$][Fe_2(μ-Se_2)$(CN)_2(CO)_4$] ([K_2(18-crown-6)$_2$][**1**]).

	[K_2(18-crown-6)$_2$][**1**]
Identification code	2374074
Empirical formula	$C_{32}H_{51}Fe_2K_2N_3O_{16}Se_2$
Formula weight	1081.57
Temperature	100(2) K
Wavelength	0.71073 Å
Crystal system	Monoclinic
Space group	$P2_1/c$
Unit cell dimensions	a = 21.2360(9) Å
	b = 8.4240(4) Å
	c = 24.7943(10) Å
Volume	4427.4(3) Å^3
Z	4
Density (calculated)	1.623 Mg/m^3
Absorption coefficient	2.555 mm^{-1}
F(000)	2200
Crystal size	0.151 x 0.072 x 0.045 mm^3
Theta range for data collection	2.454 to 25.409°.
Index ranges	-25 <= h <= 25, -10 <= k <= 10, -29 <= l <= 29
Reflections collected	70639
Independent reflections	69322 [R(int) = ?]
Completeness to theta = 25.242°	93.9 %
Absorption correction	Semi-empirical from equivalents
Max. and min. transmission	0.745210 and 0.663055
Refinement method	Full-matrix least-squares on F^2
Data / restraints / parameters	69322 / 38 / 535
Goodness-of-fit on F^2	1.061
Final R indices [I>2sigma(I)]	R1 = 0.0622, wR2 = 0.1223
R indices (all data)	R1 = 0.0873, wR2 = 0.1307
Extinction coefficient	n/a
Largest diff. peak and hole	0.899 and -0.648 e.Å^{-3}

References

(1) Rao, G.; Yu, X.; Zhang, Y.; Rauchfuss, T. B.; Britt, R. D. Fully Refined Semisynthesis of the [FeFe] Hydrogenase H-Cluster. *Biochemistry* **2023**, *62*, 2868-2877.

(2) Calvo, R.; Abresch, E. C.; Bittl, R.; Feher, G.; Hofbauer, W.; Isaacson, R. A.; Lubitz, W.; Okamura, M. Y.; Paddock, M. L. EPR study of the molecular and electronic structure of the semiquinone biradical Q_a^{-}•Q_b^{-}• in photosynthetic reaction centers from *Rhodobacter sphaeroides*. *Journal of the American Chemical Society* **2000**, *122*, 7327-7341.

(3) Stoll, S.; Schweiger, A. EasySpin, a comprehensive software package for spectral simulation and analysis in EPR. *J. Magn. Reson.* **2006**, *178*, 42-55.

(4) Kowal, A. T.; Adams, M. W.; Johnson, M. K. Electron paramagnetic resonance studies of the low temperature photolytic behavior of oxidized hydrogenase I from Clostridium pasteurianum. *J. Biol. Chem.* **1989**, *264*, 4342-4348.

(5) Patil, D. S.; Czechowski, M. H.; Huynh, B. H.; LeGall, J.; Peck, H. D.; DerVartanian, D. V. A reversible effect of low carbon monoxide concentrations on the EPR spectra of the periplasmic hydrogenase from Desulfovibriovulgaris. *Biochem. Biophys. Res. Commun.* **1986**, *137*, 1086-1093.

(6) Berggren, G.; Adamska, A.; Lambertz, C.; Simmons, T. R.; Esselborn, J.; Atta, M.; Gambarelli, S.; Mouesca, J. M.; Reijerse, E.; Lubitz, W.; et al. Biomimetic assembly and activation of [FeFe]-hydrogenases. *Nature* **2013**, *499*, 66-69.
